# Supplementary material for: Factors shaping the abundance and diversity of the gut archaeome across the animal kingdom
Source: Nat Commun. 2022 Jun 10;13:3358. doi: 10.1038/s41467-022-31038-4 (PMC9187648; doi:10.1038/s41467-022-31038-4)
Supplement: Supplementary file 1 — Supplementary Information [file 41467_2022_31038_MOESM1_ESM.pdf]

Supplementary Information

Factors shaping the abundance and diversity of the gut archaeome across the animal kingdom

Courtney M. Thomas[1,2], Elie Desmond-Le Quemener[3], Simonetta Gribaldo[1] and Guillaume Borrel[1,*]

* Corresponding author: guillaume.borrel@pasteur.fr

The file contains:

Supplementary Text
Supplementary Tables 1 to 5
Supplementary Figures 1 to 19
Supplementary References

Supplementary text

Influence of other factors on archaeal community structure and abundance

The geographic origin of the samples only explained a minor part of the variance (Table 1) and different individuals from a same species generally have close archaeal composition profiles (Supplementary Figure 16). Our dataset did not allow us to determine if captivity significantly influences the structure of the archaeal community as we would need to compare wild and captive animals belonging to the same species.

Other factors such as the absolute abundance of archaea (copies of 16S per gram of feces) and archaea:bacteria ratio as determined by qPCR did significantly impact the structure of the mammalian archaeome (Table 1). The abundance of archaea and bacteria are correlated (Figure 2c). Animal body mass and stomach pH were not significantly related to the abundance of archaea in these animals.

Absolute abundance of archaeal lineages in host lineages

Using lineage specific qPCR primers, we determined that the *Methanobacteriales* were the most prevalent methanogens detected (36% of species), followed by the *Methanomassiliicoccales* (28.3%%), the *Methanimicrococcus* (26%) and finally the *Methanomicrobiales* (23.8%). The *Thaumarchaeota* were detected in (46.1%) of species sampled. The *Methanobacteriales* and *Methanomassiliicoccales* were not detected by qPCR in the sampled Invertebrates (Insecta, Malacostraca, Mollusca, and Gastropoda), and the Actinopterygii with group specific primers, potentially because they were below the detection limit. The *Thaumarchaeota*, *Methanomicrobiales*, and *Methanimicrococcus*, were detected in at least one species from each animal class (Supplementary Figure 8). We did not identify any significant relationship between the absolute abundance of archaea and the animal phylogeny through the Moran Index.

Methane metabolisms

Overall, 45.3% of animals hosted a methanogen community dominated ($\geq$60% of the reads) by CO_2-reducing hydrogenotrophic methanogens, while 22% of the hosts have a methanogen community dominated by methyl-reducing hydrogenotrophs. In several animal lineages, the intestinal methanogenic community is consistently dominated by either methyl-reducing hydrogenotrophic methanogens or CO_2-reducing hydrogenotrophic methanogens. In the Perissodactyla/Cetartiodactyla, CO_2-reducing hydrogenotrophic methanogens represent 86.9% of the reads on average. Methyl-reducing methanogens are the dominant or codominant methanogens in 15 out of the 24 Primates species. In the Lemuridae methyl-reducing hydrogenotrophic methanogens represent 68.7% of the reads on average. While in the Hominidae, CO_2-reducing hydrogenotrophic methanogens represent 76% of reads on average (Figure 3e).

Details on the dominant archaeal orders in the animal gut

Methanobacteriales represents the largest fraction of the reads (43.1 % of reads, 491 ASVs out of 1307; Figure 2a). The *Methanobacteriales* were the most common archaea in the Hominidae, Cetartiodactyla, Rodentia, and Lagomorpha and were found in high relative abundance in two large flightless birds– the Lesser Rhea and Greater Rhea (Figure 3d). The majority of the *Methanobacteriales* reads (79.9%) were annotated as *Methanobrevibacter* (Figure 4). More than 80% of the *Methanobacteriales* reads were found in mammals and these

reads formed several clades in the reference tree that are enriched in specific host orders or host diet (Figure 4). Other mammalian-derived *Methanobrevibacter* ASVs tended to form clades according to the taxonomic affiliation of their host, Perissodactyla (two clades), Cetartiodactyla (two clades), Perissodactyla/ Cetartiodactyla mixed (one clade), Primates (three clades), and Rodentia (one clade) (Figure 4). Compared to other mammalian orders with more than five sampled species, there were no large monophyletic clades of *Methanobrevibacter* strongly enriched in sequences from Carnivora.

We identified fewer ASVs in other *Methanobacteriales* genera. Indeed only 14.8% and 4.5% of all the *Methanobacteriales* ASVs were annotated as *Methanosphaera* and *Methanobacterium*, respectively. Within the *Methanosphaera*, there are three distinct clades of ASVs from Cetartiodactyla/Perissodactyla, Primates, and Carnivora. *Methanobacterium* was also detected in mammals from the orders Carnivora, Pilosa, and Cingulata (Figure 4). A large proportion of the most prevalent *Methanobacteriales* ASVs (found in $\geq$5% of mammalian species) are highly specific of the ruminant Cetartiodactyla (Supplementary Figure 14).

<u>*Methanomassiliicoccales*</u> represents the second largest fraction of the reads (19.1% of the reads, 206 ASVs; Figure 2a). Lemuridae have a high relative abundance of *Methanomassiliicoccales* compared to closely related animal groups (Figure 3d). Within the *Methanomassiliicoccales* included in our reference tree (those representing at least 1% of the archaeome in one host), most of the ASVs (93/98) cluster with the *Ca*. Methanomethylophilaceae previously referred as a "host-associated clade" [Sup1–3], as do a majority of the associated reads (91.7%). The majority (69.4%) of the *Methanomassiliicoccales* ASVs were also mammal-derived (Supplementary Figure 5). ASVs from the Perissodactyla cluster with sequences that were obtained from other studies of closely related Cetartiodactyla. There is one clade that is enriched in ASVs derived from Primates. ASVs from two reptilian orders, Squamata (carnivorous) and Testudines also appear in the tree. Interestingly, a majority of reads attributed to reptiles came from the *Methanomassiliicoccaceae* / the "free-living clade" [Sup1–3] (Supplementary Figure 5). Like the *Methanobacteriales*, the *Methanomassiliicoccales* are largely absent in the bony fishes and invertebrates which is supported by qPCR data (Supplementary Figure 8).

<u>*Nitrososphaerales*</u>/*Nitrososphaeraceae* (phylum *Thaumarchaeota*) is the third lineage that comprises the largest number of reads (15.7% of the reads, 267 ASVs; Figure 2a). However, their absolute abundance is generally around 10^6 copies per gram of feces or less (Figure 2c). Thus, they generally dominate the archaeome of animals with a low concentration of total archaea such as the invertebrates, some birds, and the order Carnivora (Figure 3b & 3d; Figure 2d). *Nitrososphaerales* ASVs represent 93% and 90% of *Thaumarchaeota* ASVs and reads, respectively. This order was previously identified in humans and apes [Sup4] (Supplementary Figure 6). Unlike the methanogenic orders, there are no phylogenetic clades that appear in one type of host in the *Thaumarchaeota* (Supplementary Figure 6). But surprisingly, several ASVs are enriched in both Carnivora and Gastropoda (Supplementary Figure 17).

<u>*Methanomicrobiales*</u> are less common than the *Methanobacteriales* and *Methanomassiliicoccales* (13.7% of the reads, 177 ASVs; Figure 2a), but they generally occur at high absolute abundance and are the dominant archaea in a third of all reptile species and in almost all existing species of the three Perissodactyla families (Equidae, Tapiridae, Rhinocerotidae), the only exception being the Malayan Tapir (Figure 3d). We found that 99.7%

of ASVs from this archaeal order cluster with *Methanocorpusculum* reference sequences while none of the ASVs cluster with *Methanomicrobium* (Supplementary Figure 4). ASVs from *Methanocorpusculum* gather into two main clades. One clade has ASVs from a variety of mammalian orders –*i.e.*, Rodentia, Diprotodontia, Carnivora, Pilosa, and Primates– as well as ASVs derived from amphibians, invertebrates, reptiles (Squamata), and birds. In this clade, three ASVs from Rodentia are well separated from the others and are only present in members of this animal order. The second large clade subdivides into several subclades each of which is composed of sequences with a strong specificity for Testudines, Perissodactyla, and Cetartiodactyla (Supplementary Figure 4). The branching of ASVs from Testudines among ASVs from Perissodactyla (and the associated paraphyly of the ASVs from Perissodactyla) could be due to phylogenetic biases.

Methanosarcinales are sparsely distributed throughout animal species, with a high relative abundance in some reptiles and insect-eating mammals. In terms of number of reads, a single genus – *Methanimicrococcus* was the fourth most abundant archaea (Figure 2a). This order of methanogens had the lowest level of diversity with just 81 ASVs. These are spilt between to genera, *Methanimicrococcus* (55 ASVs) and *Methanosarcina* (26 ASVs) (Supplementary Figure 18). However, 92.7% of the *Methanosarcinales* reads were attributed to *Methanimicrococcus*, while *Methanosarcina* only accounted for 7.3%. The genus *Methanimicrococcus* has largely been associated with the digestive tract of termites and cockroaches [Sup5–11], but recently was shown to form two distinct clades -one of sequences obtained from insects and another from mammals [Sup12]. One ASV that clustered with insect-derived *Methanimicrococcus* reference sequences came from a frog and a toad possibly indicating that these amphibians acquired this methanogen from their prey. Similarly, we found that several ASVs from other animals that feed on invertebrates such as the long-spine squirrel fish, common gull, Eurasian coot, Great spotted woodpecker all cluster within the same clade of the insect-enriched reference sequences of *Methanimicrococcus*. The largest number of reads we obtained for the *Methanimicrococcus* came from animals whose primary diet is invertebrates (Figure 3d). Most *Methanimicrococcus* mammalian-derived ASVs fall within the previously proposed mammal clade [Sup12] (Supplementary Figure 18).

Cooccurrence analysis

It was not possible to run a cooccurrence analysis on all animals because of the sampling distribution and the number of ASVs (clustered into OTUs) shared between groups. However, it was possible to analyze several lineages of animal for which enough samples were collected and there was enough overlap between ASVs. This analysis was thus run on all mammal samples and independently on mammalian orders, i.e., Primates, Perissodactyla/Cetartiodactyla and Carnivora. Further, because few studies have been completed targeting the ecology of intestinal archaea in birds and reptiles and we had enough samples for robust statistical analyses, we also ran a cooccurrence analysis on each of these two classes of animals.

Mammals. Across mammals we identified 620 OTUs that were present in $\geq$10% of all mammal species. There were six archaea-bacteria relationships identified in both co-occurrence algorithms, four of which were between methanogens and *Clostridiales* (Supplementary Data 2). In addition to its cooccurrence with an OTUs closely related to *Lachnospira pectinoschiza*, *Ca.* Methanomethylophilaceae OTUarc_11 was also found to be significantly correlated to the

presence of a bacterial-OTU belonging to the family *Muribaculaceae* (phylum *Bacteroidetes*) which was particularly abundant in the Cingulata. A BLAST of this bacterial OTU sequence did not result in any significant results. This family of bacteria was previously identified to be a dominant member of the rodent intestinal microbiome and to have the capacity to degrade pectin [Sup13,14], likely forming methanol. However, the lack of a clear annotation of this bacterial OTU leaves the details of the relationship between these archaea and bacteria unclear. The *Ca.* Nitrosocosmicus OTUarc_45 (including ASV4) - the most widespread *Thaumarchaeota* in our dataset - was significantly linked to *Solobacterium* which was isolated from human feces [Sup15]. The cooccurrence of these two OTUs is also observed when only including Primates samples in the analysis.

Ungulata: Perissodactyla and Cetartiodactyla. The highest number of archaeal-bacterial relationships was observed in the Ungulata (Perissodactyla and Cetartiodactyla; Supplementary Data 2) with 17 positive associations and seven negative associations. These orders also have the highest concentration of archaea on average ($3.3x10^8$ 16S rRNA gene copies/gram of feces), as well as some of the highest diversity of archaea. Nine positive relationships we identified were between Archaea and members of the *Clostridiales*, four were between Archaea and members of the *Bacteroidetes*, and the other four were between Archaea and either *Melainabacteria*, *Patescibacteria*, or *Tenericutes* (Supplementary Data 2).

Two *Christensenellaceae* OTUs (OTUbac_67 and OTUbac_2376) were negatively correlated to methanogen OTUs: a *Methanobrevibacter* OTUarc_20 (corresponding to *M. ruminantium*) and *Ca.* Methanomethylophilaceae OTUarc_189; Supplementary Data 2). This is surprising as *Christensenellaceae* have previously been shown to be positively correlated with *Methanobrevibacter smithii* in the human intestine and some of its representatives support the growth of this methanogen [Sup16]. However, a third *Christensenellaceae* OTU (OTUbac_503) was positively correlated to *Methanobrevibacter* OTUarc_33 (corresponding to *Methanobrevibacter wolinii*; Supplementary Data 2). *Methanobrevibacter* OTUarc_20 and *Ca.* Methanomethylophilaceae OTUarc_189 were also negatively correlated with a *Ruminococcaceae* OTU and a Patescibacteria OTU, respectively.

Primates. All the archaea-bacteria associations we identified in primates were between archaea and *Firmicutes*. The positive association of a pectin-degrading bacterium *Lachnospira* (OTUbac_2345) and *Ca.* Methanomethylophilaceae OTUarc_11, found when considering all Mammals, was also identified when the microbial community of primates was analyzed independently. Although the edge stability was slightly below the cut off threshold of 0.5, all the other metrics indicate the significance of this relationship (edge stability = 0.45, p = 0.05, rho =0.42). Two CO_2-reducing hydrogenotrophic methanogens the *Methanobrevibacter* and *Methanocorpusculum* were also found to be positively correlated to members of uncharacterized *Ruminococcaceae* one of the most abundant family of bacteria in the gut of mammals [Sup17].

Carnivora. Carnivora members host a unique community of intestinal archaea among mammals – a low concentration of archaea, and a number of them have an archaeome dominated by *Thaumarchaeota* - thus we also performed an individual cooccurrence analysis on this group of mammals. OTUs belonging to the *Methanobacteriales* and *Nitrososphaerales* are significantly correlated to several members of the *Firmicutes* and one *Proteobacteria* in

the Carnivora (Supplementary Data 2). *Methanobacterium* (OTUarc_325 – 95% similarity to *Methanobacterium formicicum*) and *Methanobrevibacter* (OTUarc_20 – 98% similarity to *M. olleyae/ruminantium*) - were positively associated to OTUs from the *Firmicutes* like *Lachnospiraceae* (*Blautia* and Tyzzerella_4, respectively). Further, we found that *Methanobrevibacter* was positively correlated to the presence of a *Lactobacillus* OTU (Supplementary Data 2). A *Ca.* Nitrosocosmicus OTU (*Thaumarchaeota*) was positively linked to a *Staphylococcus* OTU (Firmicutes), but negatively linked to *Enterobacteriaceae* OTU corresponding to *Escherichia/Shigella* (*Proteobacteria*). The cooccurrence between a dominant intestinal bacterium and a newly identified genus of the intestinal archaea warrants further investigation.

<u>Aves and Reptiles.</u> In Aves, no archaea-bacteria relationships shared between the SparCC and SPIEC-EASI approaches (Supplementary Data 2). However, the SparCC approach, identified one significant archaea-bacteria relationship between a Methanosphaera OTU and a *Clostridiales* (Clostridium *sensu stricto* 1); and the SPIEC-EASI approach identified 57 relationships between archaea and bacteria. Interestingly this approach identified that *M. smithii* (OTUarc_1), is negatively correlated to several bacterial OTUs, including common gut bacterial groups such as *Clostridia* and *Arthrobacter* – a species previously characterized as being part of the goose core-gut microbiome[Sup18]. It is currently not clear as to why this apparently well-adapted intestinal archaea would be negatively related to common intestinal bacteria in birds. Other archaeal OTUs such as *Methanosphaera*, *Ca*. Methanomethylophilaceae, and *Nitrososphaeraceae* were positively correlated to various bacteria groups (Supplementary Data 2). For example, one of the *Nitrososphaeraceae* OTUs was positively correlated with a *Blautia* OTU. The same bacterial OTU was also negatively correlated to OTUarc_1.

Two archaeal OTUs were linked to bacteria in reptiles using both approaches. A *Methanocorpusculum* OTU is significantly positively correlated to a *Providencia* OTU (100% identical to *Providencia rettgeri*; *Enterobacteriales*) which is a common constituent of the human and reptile intestinal tract [Sup19]. Also, *Methanomassiliicoccus* is positively correlated to the Clostridiaceae_1 family (95% identical to *Clostridium cylindrosporum)*.

<u>Cooccurrence between archaeal OTUs.</u> Globally, these analyses also highlighted an absence of negative archaea-archaea relationship and several positive archaea-archaea relationships between OTUs of a same family. This was notably the case between *Methanobrevibacter* and *Methanosphaera* OTUs (*Methanobacteriaceae*) in Primates, Ungulata and overall mammals, but no association were observed between *Methanobrevibacter* OTUs or between *Methanosphaera* OTUs. However, a *Ca*. Methanomethylophilaceae OTUs was also associated to another *Ca*. Methanomethylophilaceae OTUs in Primates (Supplementary Data 2). *Nitrososphaeraceae* OTUs were positively associated to each other in mammals.

Supplementary Tables

Table S1: Percentage of ASVs (n = 1307) and total reads corresponding to characterized archaea (isolates/enriched). ASVs sequences were compared to 16S rRNA genes of type strain archaea in the SILVA Living Tree Project LTP plus additional sequences of enriched archaea whose genome was sequenced (custom database) and with the SILVA Living Tree Project LTP alone.

	**Similarity with characterized archaea at different levels**			
	**95**	**97**	**98.7***	**99**
Custom database + Silva LTP	*84.9%*	*53.9%*	*31%*	*20.4%*
% of reads with BLAST hit	*(94.5%)*	*(75.5%)*	*(52.5%)*	*(46.7%)*
Silva - Living Tree Project	*66.1%*	*35.6%*	*18%*	*11.8%*
% of reads with BLAST hit	*(74.2%)*	*(51.3%)*	*(34.5%)*	*(31.3%)*

* Proposed species level similarity of 16S rRNA genes by Yarza et al 2014

Table S2: Known metabolisms of archaeal lineages identified in the animal gut

Phylum	Class	Order	Family	Best annotation	Methane metabolism	Remark on methane metabolism	Other energetic metabolism	Relation to oxygen
Euryarchaeota	Methanobacteria	Methanobacteriales	*Methanobacteriaceae*	*Methanobacterium*	Hydorgenotrophic CO2 -reducing			Strict anaerobe
Euryarchaeota	Methanobacteria	Methanobacteriales	*Methanobacteriaceae*	*Methanobrevibacter*	Hydorgenotrophic CO2 -reducing			Strict anaerobe
Euryarchaeota	Methanobacteria	Methanobacteriales	*Methanothermobacteraceae*	*Methanothermobacter*	Hydorgenotrophic CO2 -reducing			Strict anaerobe
Euryarchaeota	Methanomicrobia	Methanocellales	*Methanocellaceae*	*Methanocella*	Hydorgenotrophic CO2 -reducing			Strict anaerobe
Euryarchaeota	Methanomicrobia	Methanocellales	*Methanocellaceae*	*Methanocellaceae*	Hydorgenotrophic CO2 -reducing			Strict anaerobe
Euryarchaeota	Methanomicrobia	Methanomicrobiales	*Methanocorpusculaceae*	*Methanocorpusculum*	Hydorgenotrophic CO2 -reducing			Strict anaerobe
Euryarchaeota	Methanomicrobia	Methanomicrobiales	*Methanoregulaceae*	*Methanoregula*	Hydorgenotrophic CO2 -reducing			Strict anaerobe
Euryarchaeota	Methanomicrobia	Methanomicrobiales	*Methanospirillaceae*	*Methanospirillum*	Hydorgenotrophic CO2 -reducing			Strict anaerobe
Euryarchaeota	Methanomicrobia	Methanomicrobiales	NA	*Methanomicrobiales*	Hydorgenotrophic CO2 -reducing			Strict anaerobe
Euryarchaeota	Methanobacteria	Methanobacteriales	*Methanobacteriaceae*	*Methanosphaera*	Hydorgenotrophic CH3 -reducing	One isolate reduces methanol by only using ethanol as electron donnor (not H_2)		Strict anaerobe
Euryarchaeota	Methanomicrobia	Methanosarcinales	*Methanosarcinaceae*	*Methanimicrococcus*	Hydorgenotrophic CH3 -reducing			Strict anaerobe
Euryarchaeota	Thermoplasmata	Methanomassiliicoccales	*Methanomassiliicoccaceae*	*Methanomassiliicoccaceae*	Hydorgenotrophic CH3 -reducing			Strict anaerobe
Euryarchaeota	Thermoplasmata	Methanomassiliicoccales	*Methanomassiliicoccaceae*	*Methanomassiliicoccus*	Hydorgenotrophic CH3 -reducing			Strict anaerobe
Euryarchaeota	Thermoplasmata	Methanomassiliicoccales	*Methanomethylophilaceae*	*Candidatus Methanogranum*	Hydorgenotrophic CH3 -reducing			Strict anaerobe
Euryarchaeota	Thermoplasmata	Methanomassiliicoccales	*Methanomethylophilaceae*	*Candidatus Methanomethylophilus*	Hydorgenotrophic CH3 -reducing			Strict anaerobe
Euryarchaeota	Thermoplasmata	Methanomassiliicoccales	*Methanomethylophilaceae*	*Candidatus Methanoplasma*	Hydorgenotrophic CH3 -reducing			Strict anaerobe
Euryarchaeota	Thermoplasmata	Methanomassiliicoccales	*Methanomethylophilaceae*	*Methanomethylophilaceae*	Hydorgenotrophic CH3 -reducing			Strict anaerobe
Euryarchaeota	Methanomicrobia	Methanosarcinales	*Methanosarcinaceae*	*Methanosarcina*	Methylotrophic / Acetoclastic / Hydrogenotrophic CH_3-reducing / Hydrogenotrophic CO_2-reducing	All species are methylotrophic and some have various combinaisons of the other methane metabolism		Strict anaerobe
Bathyarchaeia	Bathyarchaeia	Bathyarchaeia_subgroup -6	NA	*Candidatus* Termitimicrobium	None		Possibly homoacetogen / organotrophic	Strict anaerobe
Bathyarchaeia	Bathyarchaeia	Bathyarchaeia_subgroup -6	NA	*Candidatus* Termiticorpusculum	None		Organotrophic	Strict anaerobe
Euryarchaeota	Halobacteria	Halobacteriales	*Halococcaceae*	*Halococcus*	None		Organotrophic	Strict aerobe
Thaumarchaeota	Nitrososphaeria	Nitrosopumilales	*Nitrosopumilaceae*	*Candidatus* Nitrosopumilus	None		Ammonia oxydizer	Strict aerobe
Thaumarchaeota	Nitrososphaeria	Nitrosopumilales	*Nitrosopumilaceae*	*Candidatus* Nitrosotenuis	None		Ammonia oxydizer	Strict aerobe
Thaumarchaeota	Nitrososphaeria	Nitrososphaerales	*Nitrososphaeraceae*	*Candidatus* Nitrocosmicus	None		Ammonia oxydizer	Strict aerobe
Thaumarchaeota	Nitrososphaeria	Nitrososphaerales	*Nitrososphaeraceae*	*Candidatus* Nitrososphaera	None		Ammonia oxydizer	Strict aerobe
Thaumarchaeota	Nitrososphaeria	Nitrososphaerales	*Nitrososphaeraceae*	*Nitrososphaeraceae*	None		Ammonia oxydizer	Strict aerobe
Thaumarchaeota	Nitrososphaeria	Nitrosotaleales	*Nitrosotaleaceae*	*Nitrosotaleaceae*	None		Ammonia oxydizer	Strict aerobe

Table S3: Ranges of methanol concentrations and H_2 partial pressure the rumen, human colon, cockroach hindgut and marine sediments environments. Values were taken from the literature.

Nbr on Fig 7b	Environment	Type	H2 (p)	Methanol (uM)	References in sup text
1	Rumen	Gut	0.00013 – 0.064	23 - 72	sup20-22
2	Human colon	Gut	0.01 – 0.19	0.5 - 450 (avg 70)	sup23-25
3	Cockroach hindgut	Gut	0.003	10	sup9
4	Marine sediments 50 cm	Marine	1.282E-07	10	sup26
5	Marine sediments 500 cm	Marine	1.282E-07	100	sup26
6	Marine sediments	Marine	1.282E-07	10 - 120	sup27
7	Hypersaline marine sediments 10 cm	Marine	2.564E-07	1 - 8	sup28
8	Hypersaline marine sediments 50 cm	Marine	8.974E-07	4	sup28

Table S4: Primers used for quantitative PCR.

Primer Pair	Targeted Group	Sequence	Annealing temperature	Reference
**Thaum494F/ 806R**	Thaumarchaeota	GAATAAGGGGTGGGCAAGT/ GGACTACVSGGGTATCTAA	61°C	Sup 29 Sup 30
**MMB282F/ MM832R**	Methanomicrobiales	ATCGRTACGGGTTGTGGG/ CACCTAACGCRCATHGTTTAC	61.6°C	Sup 31
**Mx765F/ Mx887R**	Methanomassiliicoccales	GGGGTAGGGGTAAAATCCTG/ CGGGGTATCTAATCCCGTTT	61.2°C	Sup 32
**Methani388F/ Methani564R**	*Methanimicrococcus*	ACAATGCAGGAAACTGTG/ TAGACCMAATAAAAGCGGCTA	60.7°C	This study
**MBT857F/ MBT1196R**	Methanobacteriales	CGWAGGGAAGCTGTTAAGT/ TACCGTCGTCCACTCCTT	60°C	Sup 31
**A-934b-F/ A-1000R**	All archaea	GAATTGGCGGGGGAGCA/ GGCCATGCACYWCYTCTC	60°C	Sup 33 (modified) Sup 34
**B-357F B-531R**	All bacteria	CTCCTACGGGAGGCAGCAG CTNYGTMTTACCGCGGCTGC	65°C	Sup 35 Sup 36

Table S5: Primers used for amplicon sequencing.

Primer Pair	Targeted Group	Sequence	Annealing temperature	Cycles	References
**515F/ 806bR**	All 16S rRNA genes	*GTGYCAGCMGCCGCGGTAA / *GGACTACNVGGGTWTCTAAT	55.5°C	30 x	Sup 37 Sup 38
**344F/ 1041R**	Archaea (step 1)	ACGGGGYGCAGCAGKCGCG/ GGCCATGCACCWCCTCTC	66.6°C	20 x	Sup 39 (modified) Sup 34
**519F/ 915R**	Archaea (step 2)	*CAGCMGCCGCGGTAA/ *GTGCTCCCCCGCCAATTCCT	55.5°C	25 x	Sup 40 Sup 41

* Illumina tags were present on 515F/519F as well as 806bR/915R
Illumina F: TCGTCGGCAGCGTCAGATGTGTATAAGAGACAG
Illumina R: GTCTCGTGGGCTCGGAGATGTGTATAAGAGACAG

Figure S1

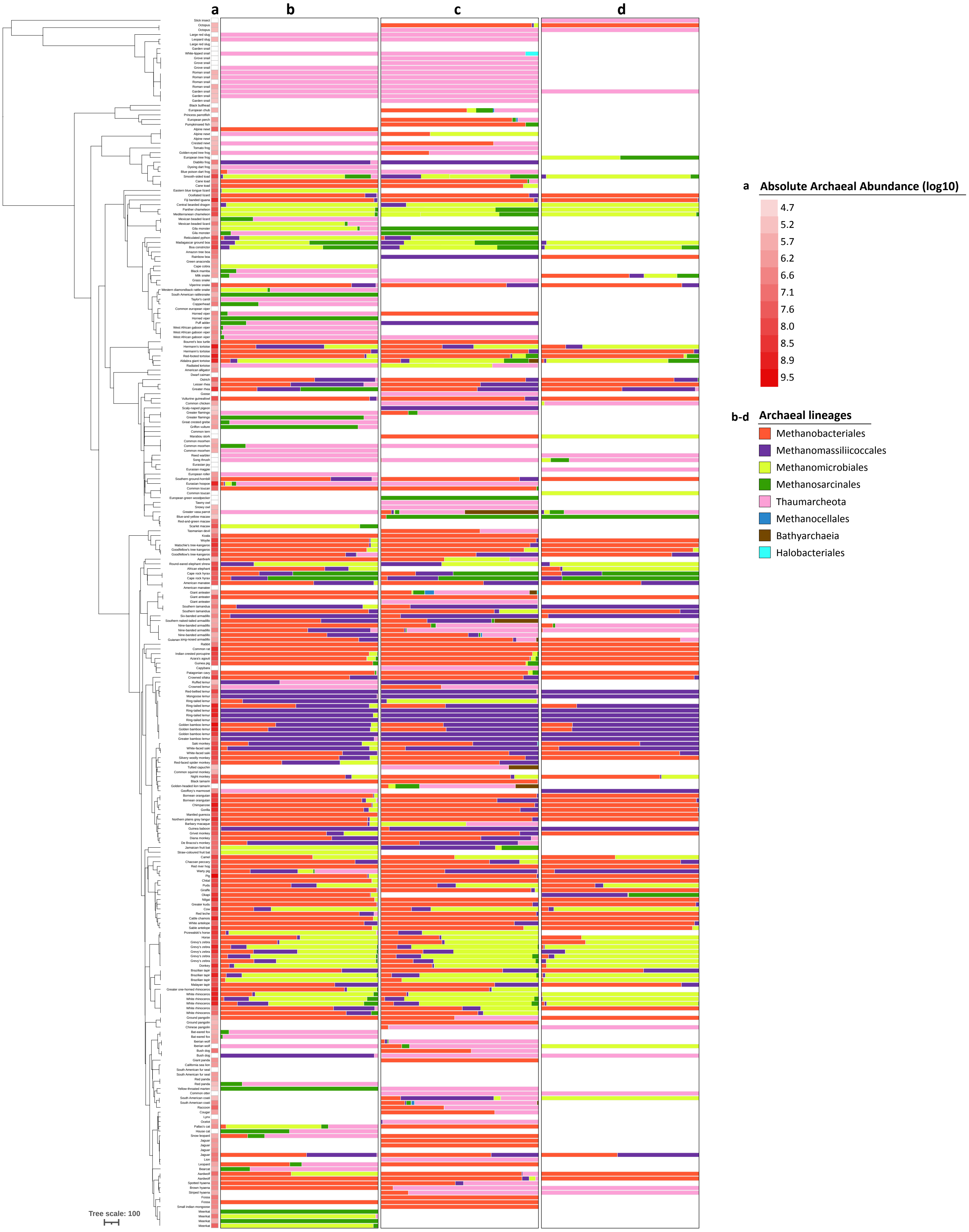

Figure S1: Comparison of the composition of the archaeal communities using three different approaches (n = 341). **a**) Abundance of archaea. **b**) Relative abundance of five archaeal lineages quantified separately using qPCR (i.e. *Methanobacteriales*, *Methanomassiliicoccales*, *Methanomicrobiales*, *Methanimicrococcus* (*Methanosarcinales*), *Thaumarchaeota*). **c**) Relative abundance of archaeal lineages in 16S rRNA gene sequencing based on archaeal-specific primers. **d**) Relative abundance of archaeal lineages in 16S rRNA gene sequencing based on prokaryotic universal primers.

Figure S2

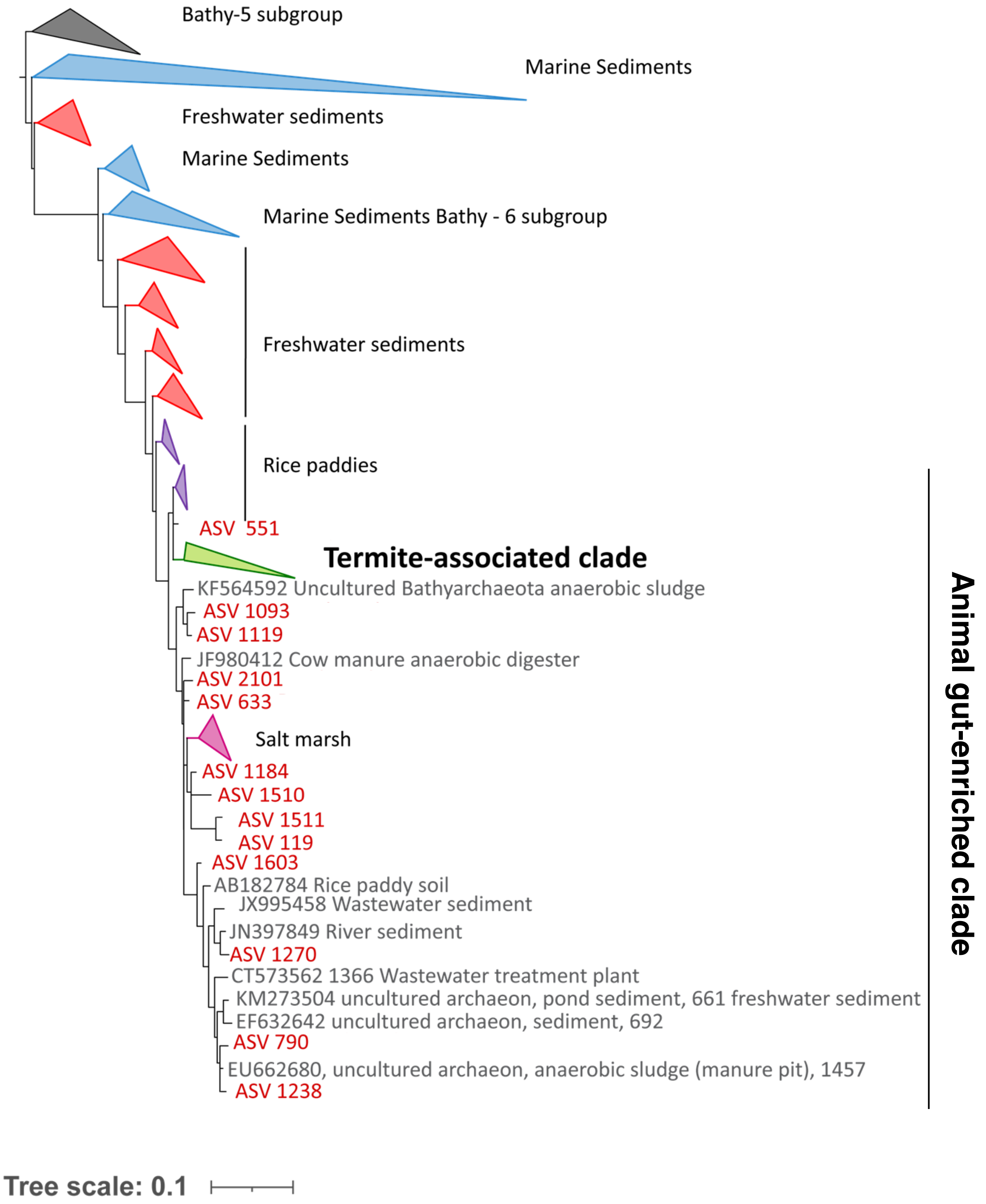

Figure S2: Close relationship between Bathyarchaeota sequences recovered from the animal gut. Sequences from this study are in red. They correspond to ASVs representing at least 1% of the gut archaeome of one animal species. Maximum-likelihood tree based on 16S rRNA gene sequences and built with GTR+G4 model. Clades are colored according to the environmnental origin of the sequences: blue, marine sediments; red, freshwater sediments; purple, rice paddies; pink, salt marshes.

Figure S3

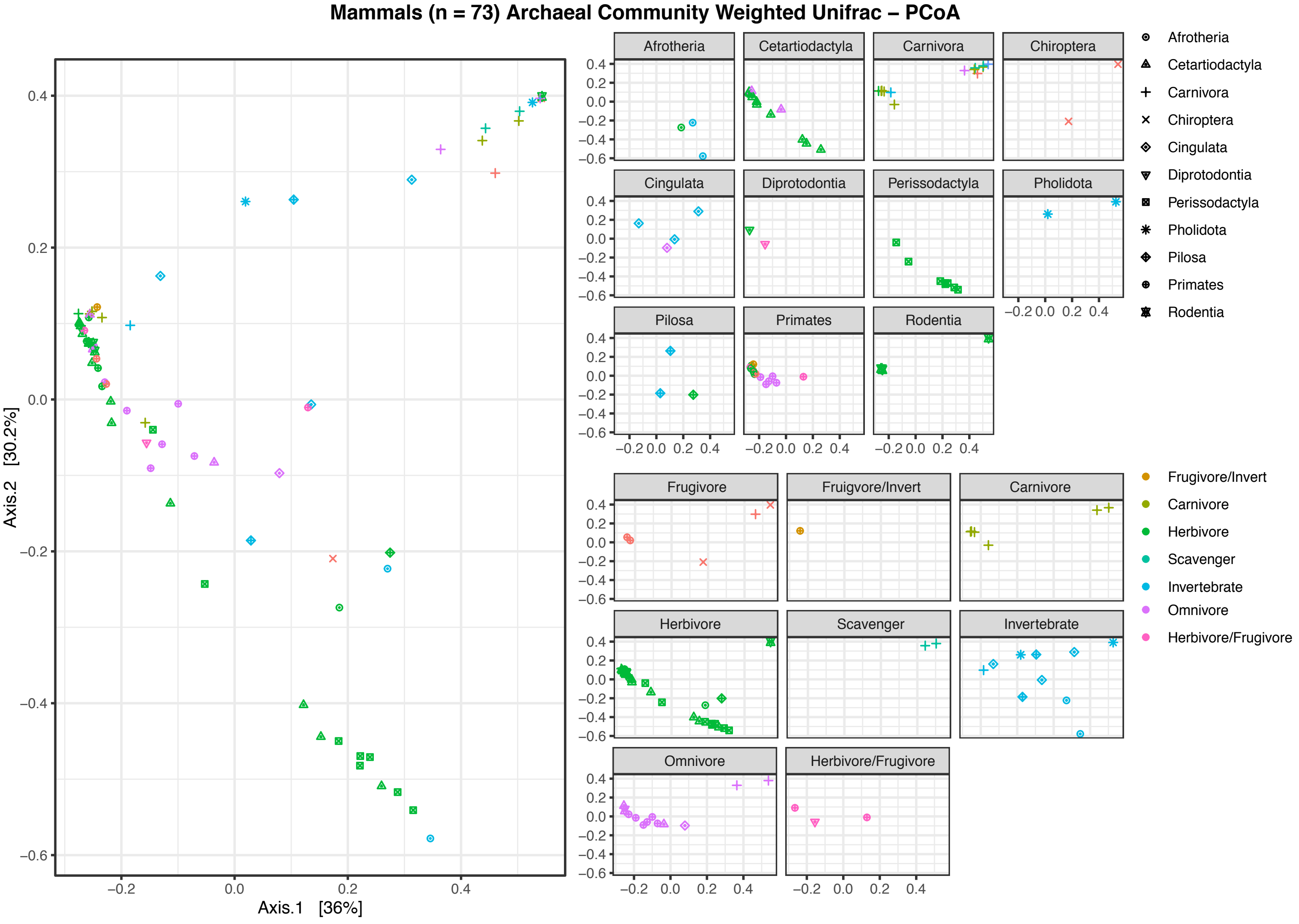

FigureS3: Weighted UniFrac analysis of the gut archaeome of Mammals, based on 73 rarefied samples with ≥3000reads.

Figure S4

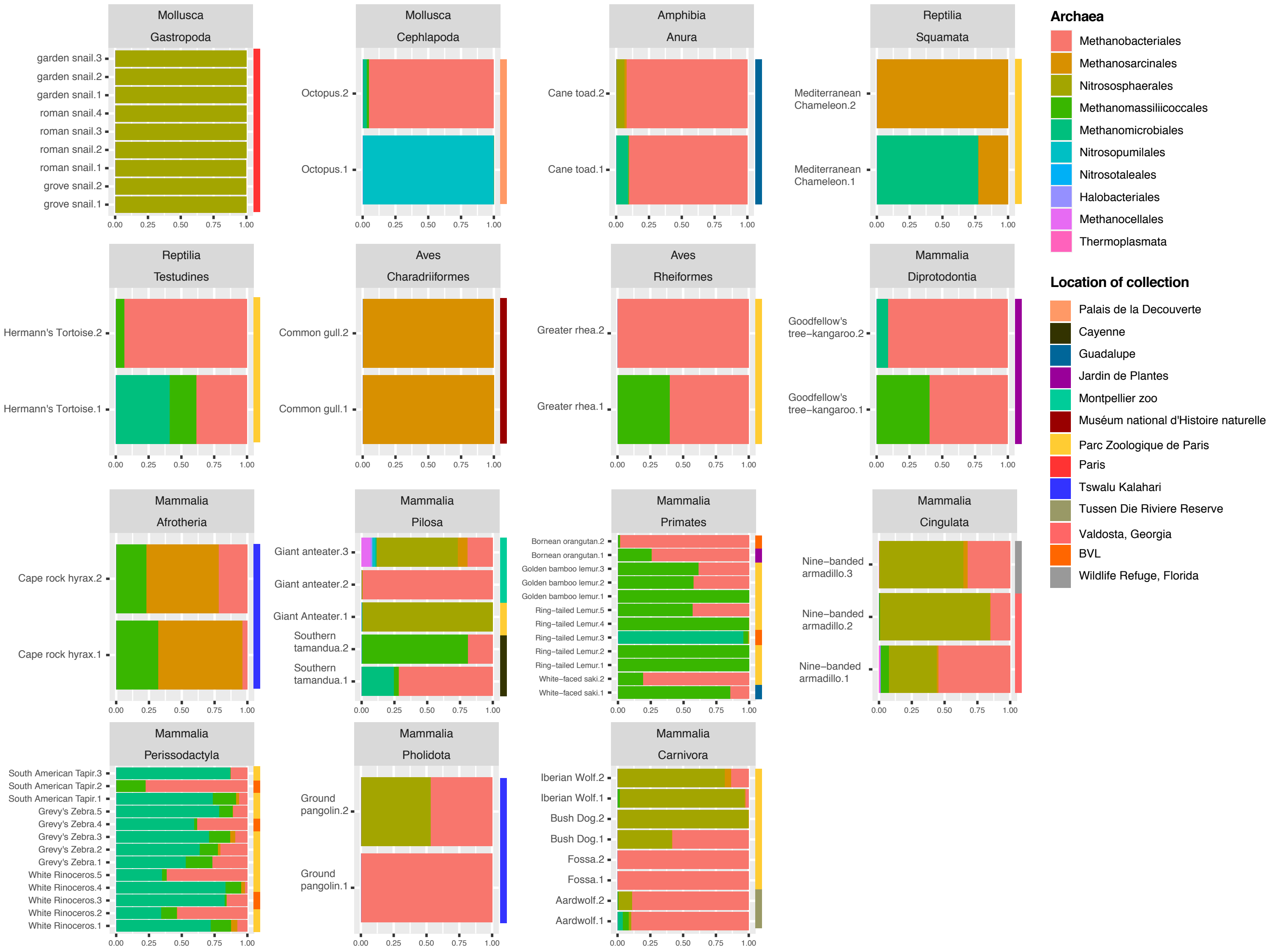

Figure S4: Intra-species variations in the composition of archaea.

Figure S5

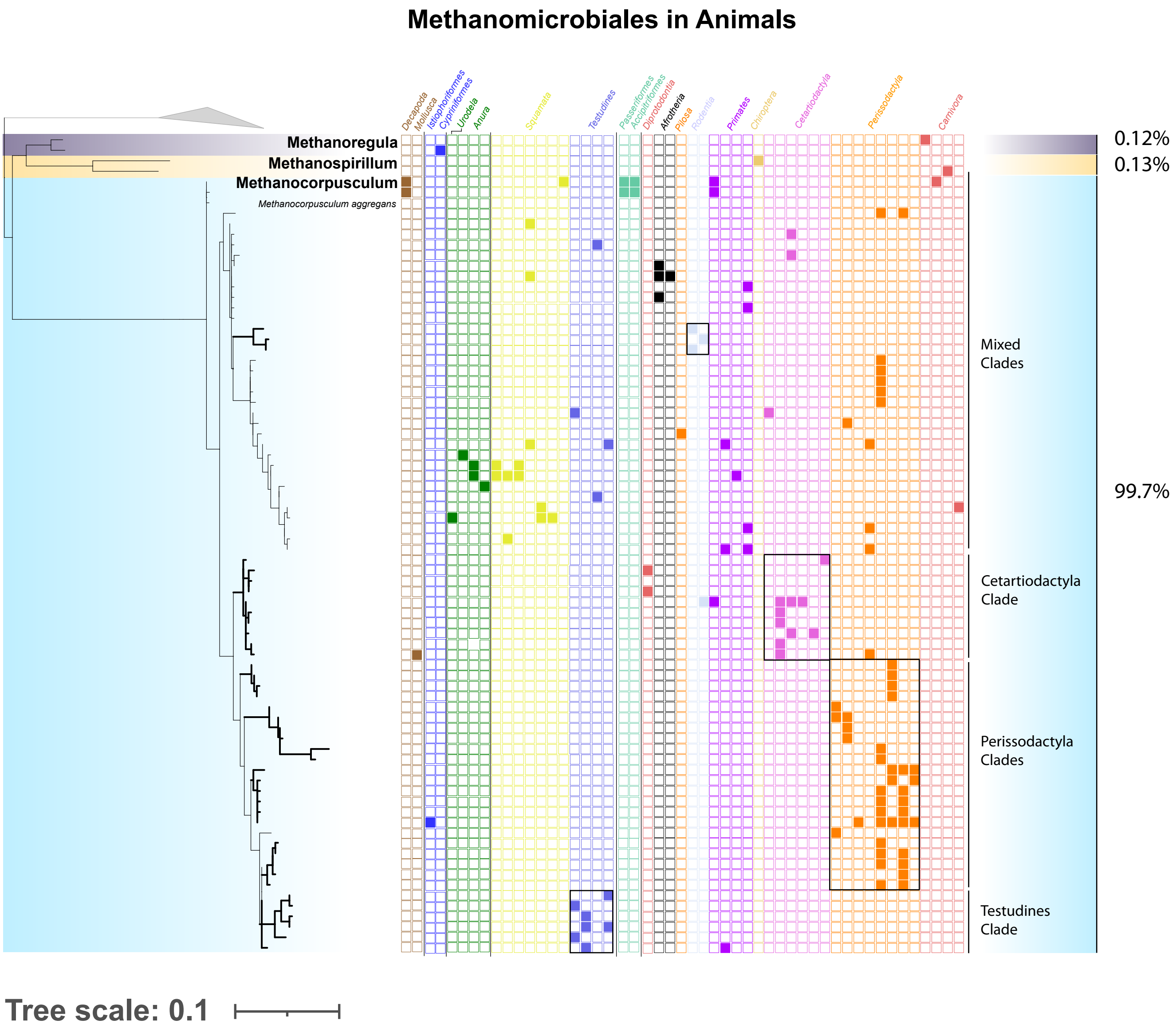

Figure S5: Distribution of Methanomicrobiales ASVs among animals. The phylogenetic tree (maximum-likelihood, GTR+G4) was built with nearly full length 16S rRNA genes sequences from literature and the ASVs sequences from this study. For clarity, the full 16S rRNA genes from literature were then removed from the tree. Only ASVs representing more than 1% of the sequences per sample were included. Presence/absence of ASVs in animals is indicated by coloured highlighted/blank squares. Animal were gathered by orders, each with a different colour, as indicated on the top. Black boxes highlight archaeal clades preferentially present in host orders. The percentages on the right represent the proportion of reads from this order that were annotated as *Methanocorpusculum* (blue background), *Methanospirillum* (yellow background) and *Methanoregula* (grey background).

Figure S6

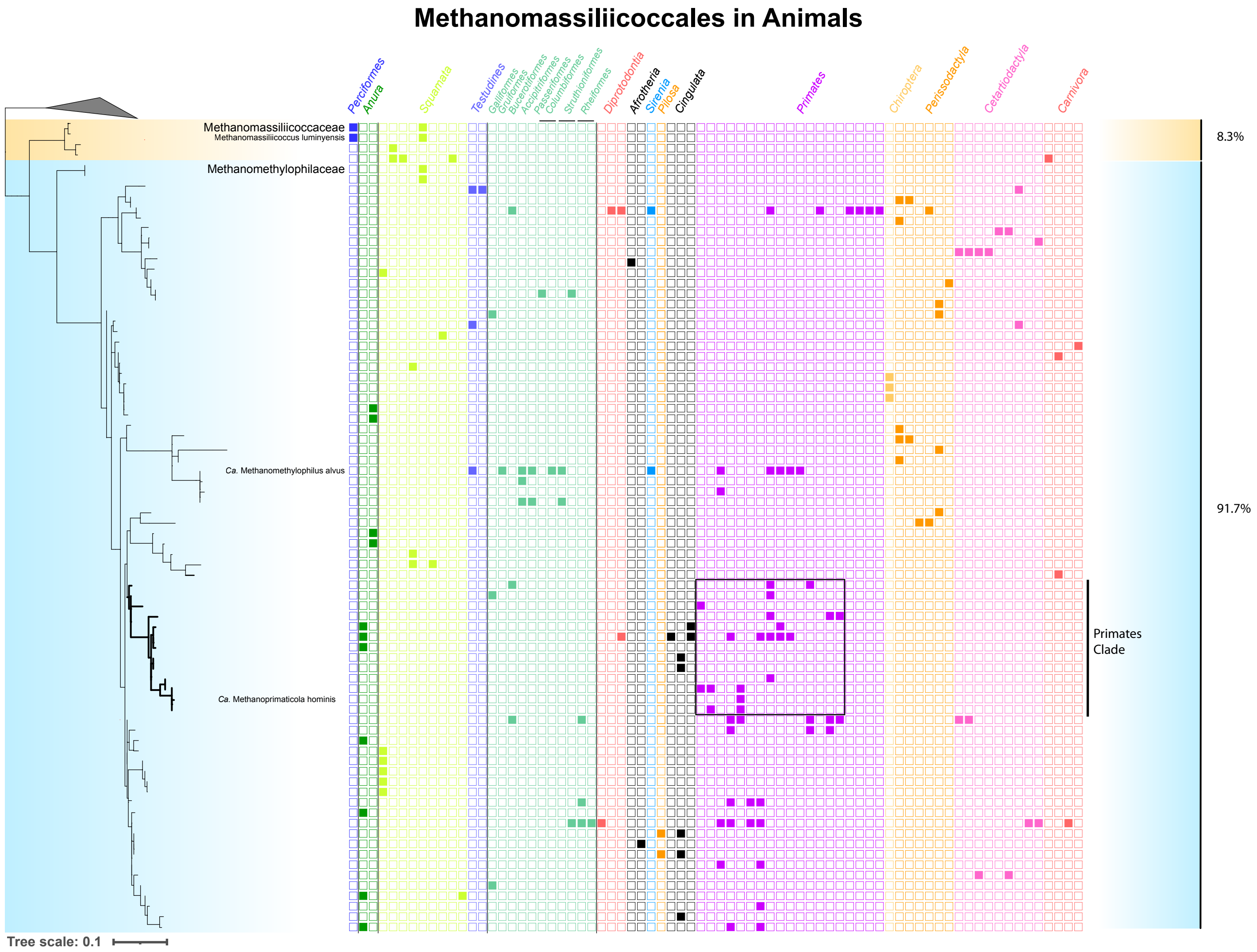

Figure S6: Distribution of Methanomassiliicoccales ASVs among animals. The phylogenetic tree (maximum-likelihood, GTR+G4) was constructed with nearly full length 16S rRNA genes sequences from literature and the ASVs sequences from this study. For clarity, the full 16S rRNA genes from literature were then removed from the tree. Only ASVs representing more than 1% of the sequences per sample were included. Presence/absence of ASVs in animals is indicated by coloured highlighted/blank squares. Animal were gathered by orders, each with a different colour, as indicated on the top. The black box highlight an archaeal clade preferentially present in Primates. The percentages on the right represent the proportion of reads from this order that were annotated as *Methanomethylophilaceae* (blue background) and *Methanomassiliicoccaceae* (yellow background).

Figure S7

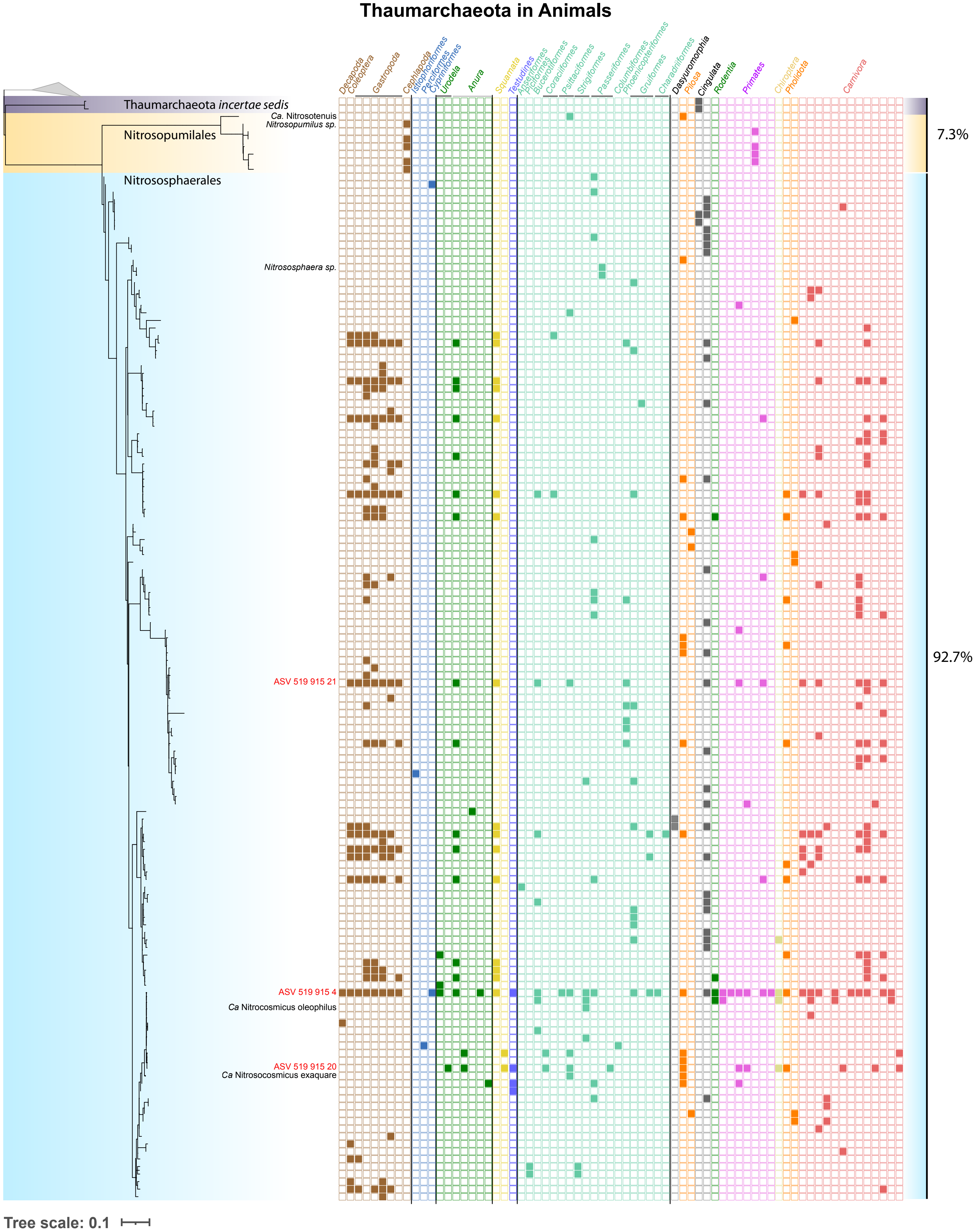

Figure S7: Distribution of Thaumarchaeota ASVs among animals. The phylogenetic tree (maximum-likelihood, GTR+G4) was constructed with nearly full length 16S rRNA genes sequences from literature and the ASVs sequences from this study. For clarity, the full16S rRNA genes from literature were then removed from the tree. Only ASVs representingmore than 1% of the sequences per sample were included. The percentages on the right represent the proportion of reads from this order that were annotated as *Nitrososphaerales* (blue background) or *Nitrosopumilales* (yellow background). The three ASVs in red on the left of the central frame are those discussed in the text.

Figure S8

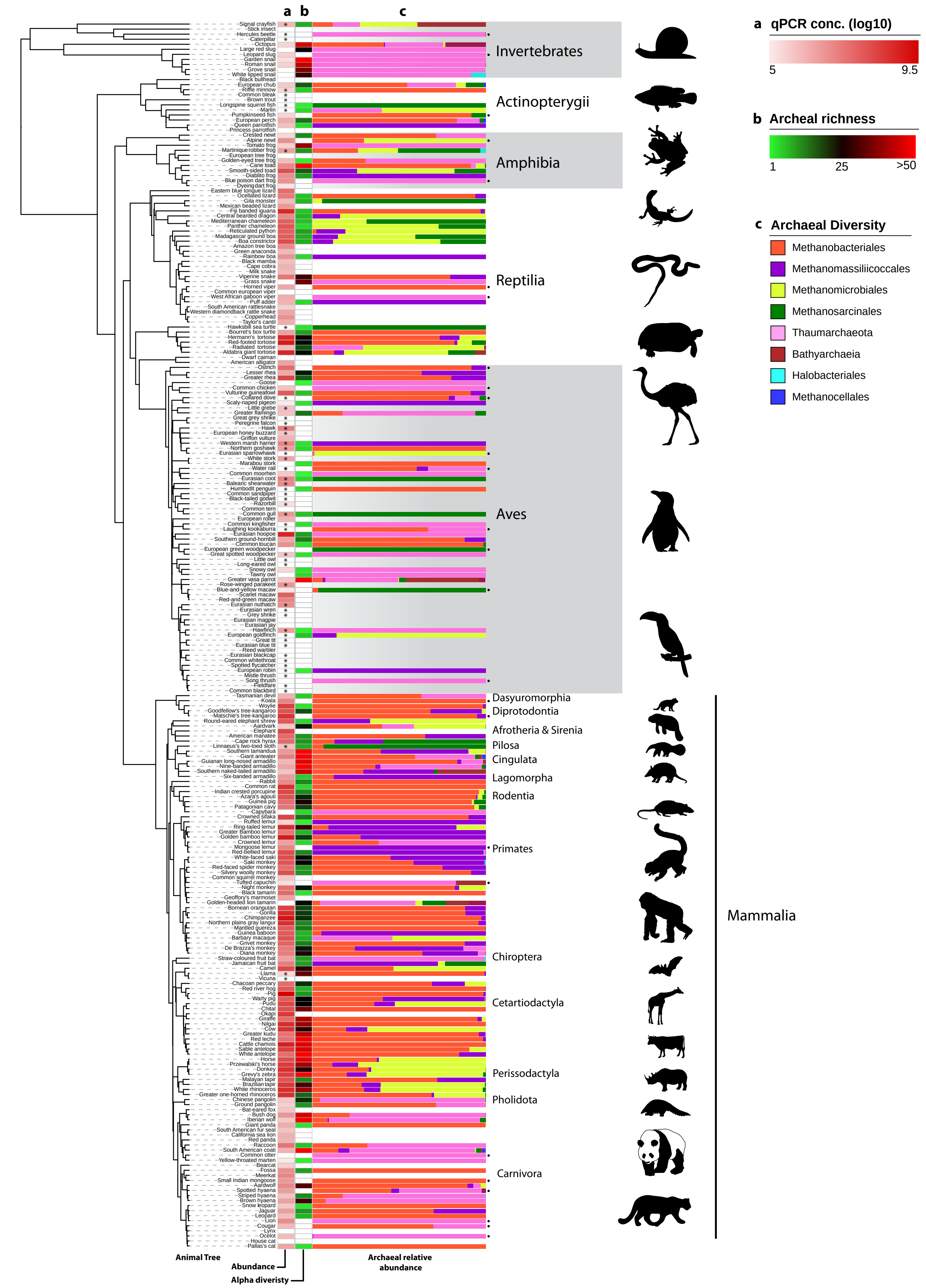

Figure S8: Archaeal diversity across animals including species for which a low number (<3000 reads) of reads or no reads were obtained. Animal species with less 3000 reads are indicated by a dote (•) on the right of the histogram. Possible underestimation of archaeal abundance in some animal species (see Figure S19) is indicated by a star (*) on the abundance heat map.

Figure S9

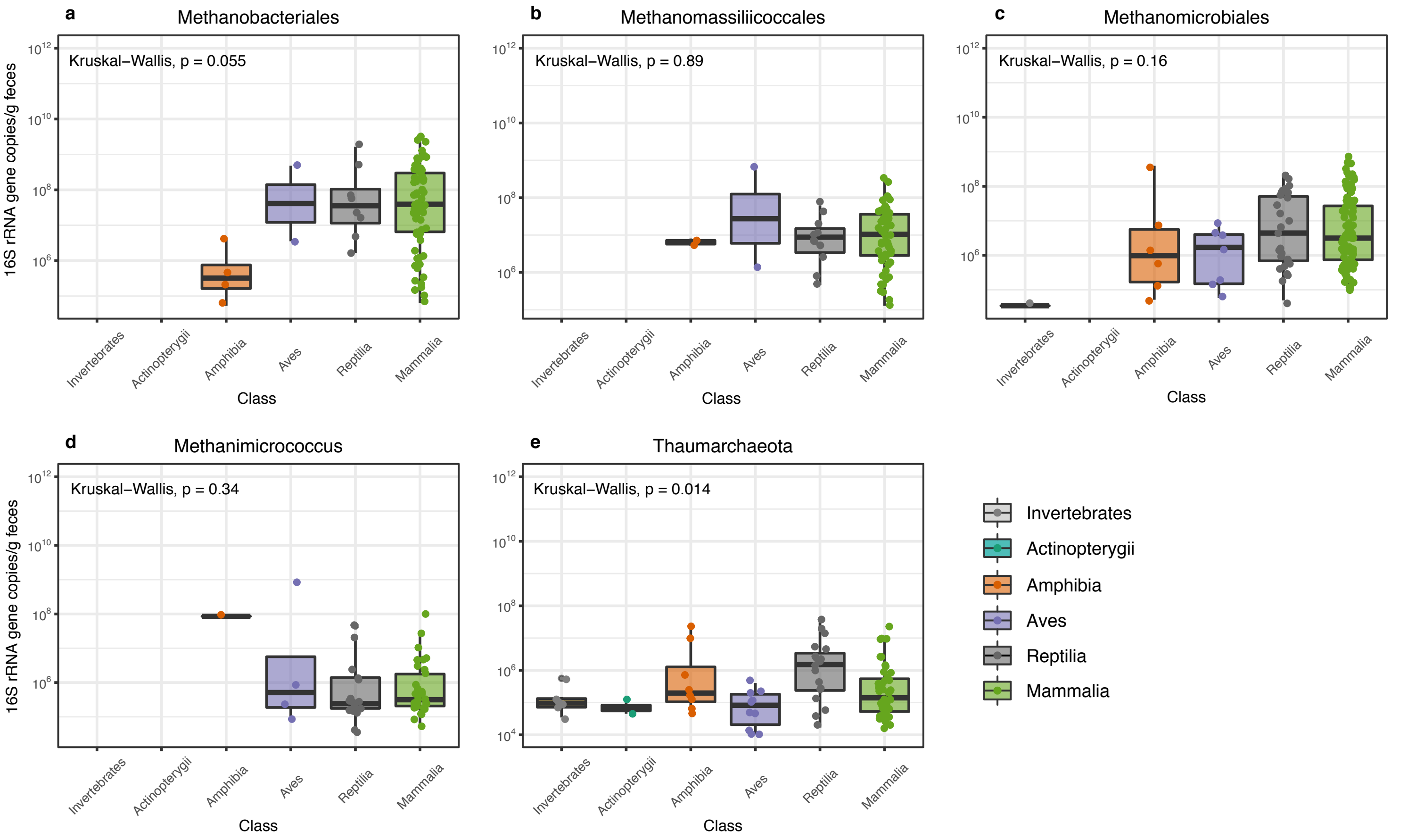

Figure S9: Absolute abundance of five archaeal lineages determined via qPCR. Significant differences across all groups were determined via the Kruskal-Wallis test, $p < 0.05$ is significant. **a**) *Methanobacteriales* (n=85); **b**) *Methanomassiliicoccales* (n=73); **c**) *Methanomicrobiales* (n=132); **d**) *Methanimicrococcu*s (n=55); **e**) *Thaumarchaeota* (n=89). Wilcoxon rank sum test with continuity correction was used to determine differences between animal classes. Two-sided Wilcoxon rank sum test with continuity correction was used to determine differences between animal classes, no signficant differences were observed. In the boxplots, the minima is the minimum value, maxima is the maximum values, center is the median and quartiles are shown by the box and whiskers, individual samples are shown as colored dots.

Figure S10

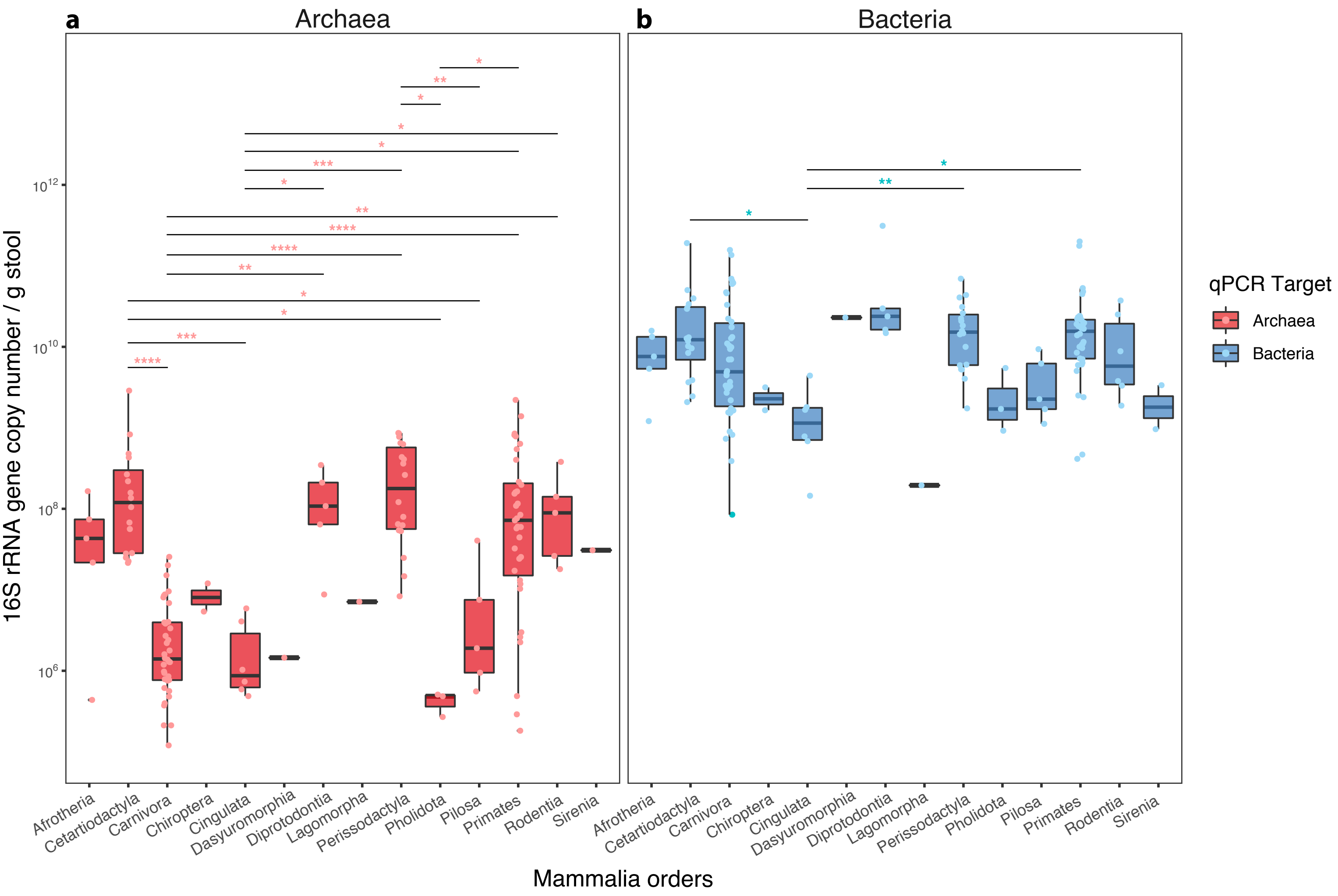

Figure S10: Absolute abundance of **a**) Archaea (n = 139) and **b**) Bacteria (n = 148) determined by qPCR in mammals (clustered by order). Animal lineages with significantly different archaeal/bacterial abundances are labeled. Two-sided Wilcoxon rank sum test *: $p < 0.05$; **: $p < 0.01$; ***: $p < 0.001$; ****: $p < 0.0001$. In the boxplots the minima is the minimum value, maxima is the maximum values, center is the median and quartiles are shown by the box and whiskers, individual samples are shown as colored dots. Exact p-values are given in Supplementary Data 4.

Figure S11

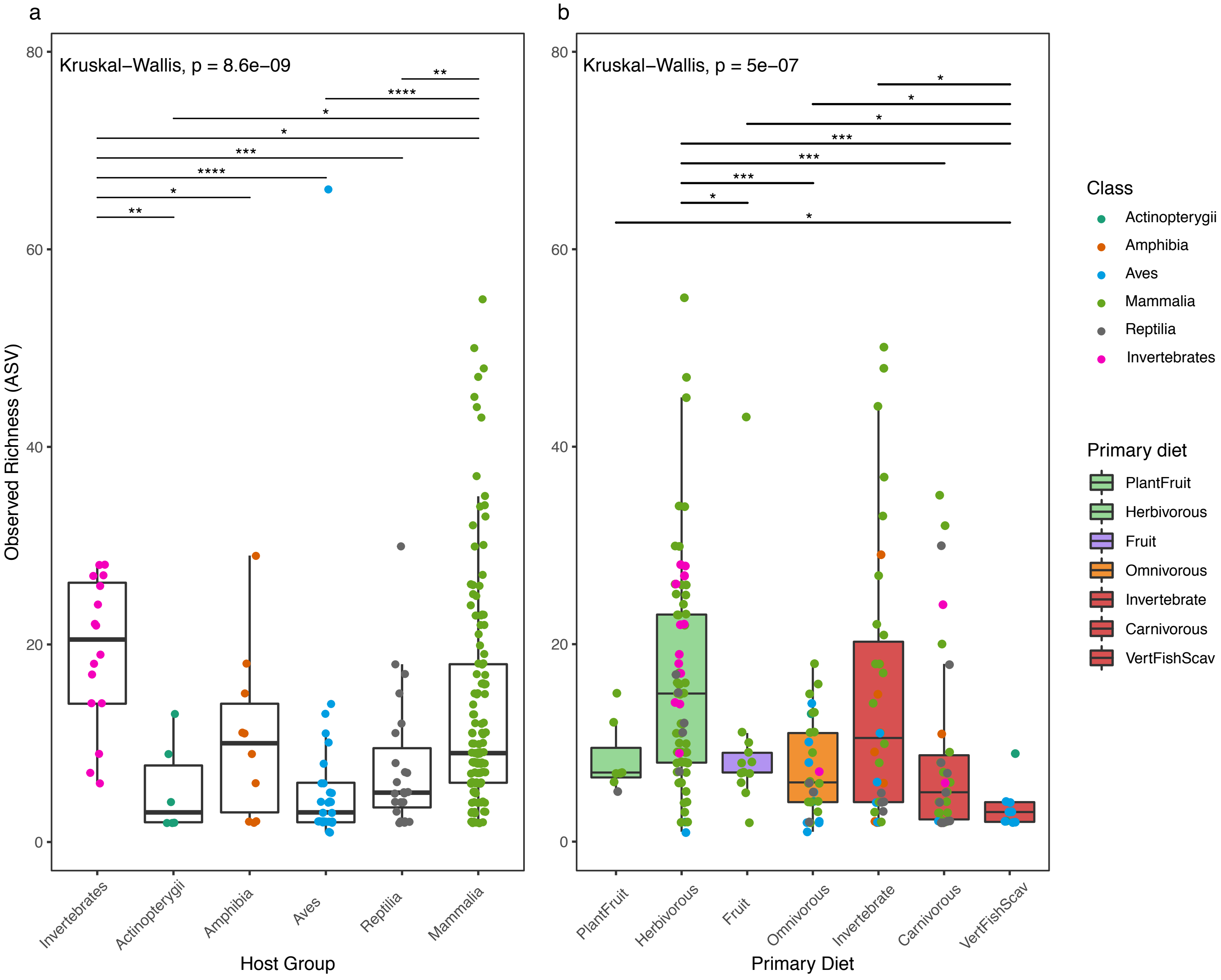

Figure S11: Observed richness of archaea in samples rarefied to 250 reads and grouped by **a**) animal classes (n=218) and **b**) diet-types (n = 197). Diet-types with fewer than 3 representatives were removed. Signficiant differnces across all groups were determined via the Kurskal-Wallis test, with $p < 0.05$ as significant. Two-sided Wilcoxon rank sum test *: $p < 0.05$; **: $p < 0.01$; ***: $p < 0.001$; ****: $p < 0.0001$. In the boxplots, the minima is the minimum value, maxima is the maximum values, center is the median and quartiles are shown by the box and whiskers, individual samples are shown as colored dots. The color of boxplot background in panel b indicates the main food type: green, leaves; purple, fruits; orange, divers; red, meat. Exact p-values are given in Supplementary Data 4.

Figure S12

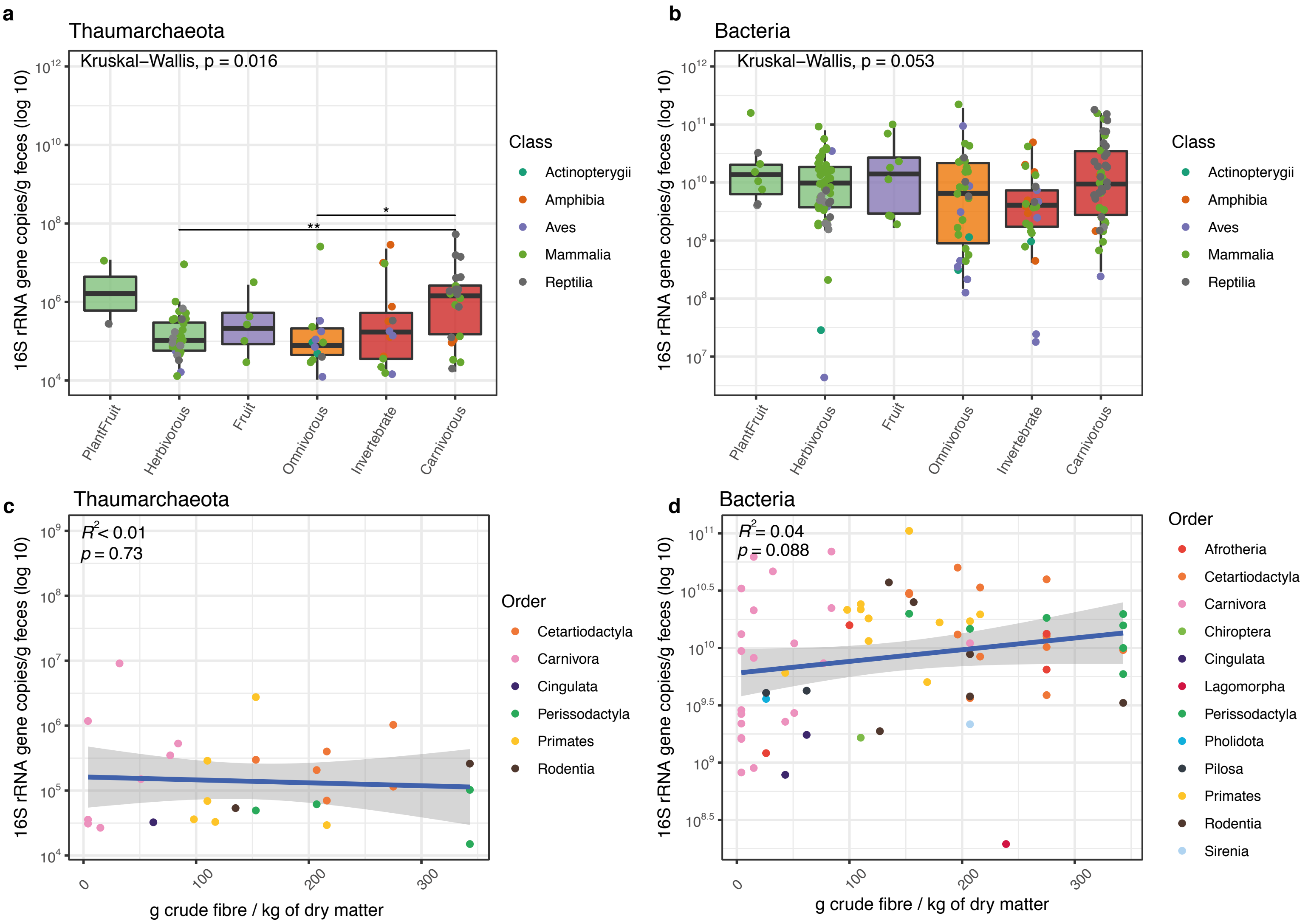

Figure S12: Influence of host diet-type, diet-fibre content on the absolute abundance of Thaumarchaeota and Bacteria. Abundance of **a**) Thaumarchaeota (n = 89) and **b**) Bacteria (n = 174) according to host diets-type. Two-sided Wilcoxon rank sum test with continuity correction was used to determine differences between diet types *: $p < 0.05$; **: $p < 0.01$. Significant differences across all groups were determined via the Kruskal-Wallis test, with $p < 0.05$ set as significant. In the boxplots, the minima is the minimum value, maxima is the maximum values, center is the median and quartiles are shown by the box and whiskers, individual samples are shown as colored dots. The color of boxplot background in panels a and b indicates the main food type: green, leaves; purple, fruits; orange, divers; red, meat. Correlation between diet-fibre content and absolute abundance of **c**) Thaumarchaeota (n = 27) and **d**) Bacteria (n = 70) in mammal species. A two-sided, squared Pearson correlation coefficient was computed to assess the relationship between values, unadjusted p-values < 0.05 were considered significant. Statistical analyses and representation of the absolute/relative abundance of methanogens were carried out on species where archaea have been detected. Grey bands around the lines (panels c and d) represent the 95% confidence interval around the linear regression model. Exact p-values of panel a) are given in Supplementary Data 4.

Figure S13

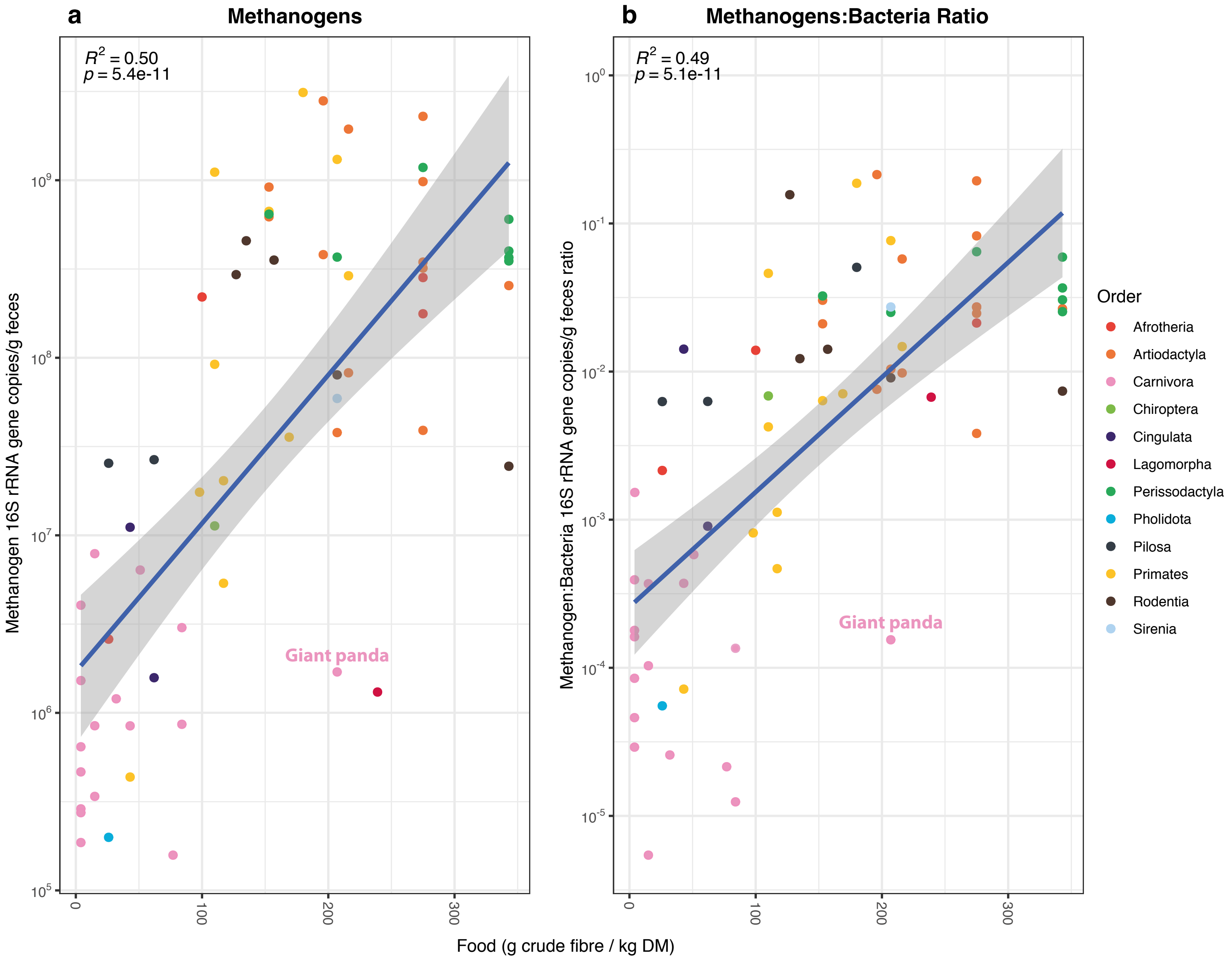

Figure S13: Correlation between diet-fibre content and **a**) absolute abundance or **b**) relative abundance of methanogens in mammal faeces (n = 65). The abundance of methanogen is determined by the sum of the abundance of Methanobacteriales, Methanomicrobiales, Methanomassiliicoccales and Methanimicrococcus determined by qPCR. The ratio of methanogens to bacteria are also based on qPCR measurements. A two-sided, squared Pearson correlation coefficient was computed to assess the relationship between values, unadjusted p-values < 0.05 were considered significant. Grey bands around the lines represent the 95% confidence interval around the linear regression model.

Figure S14

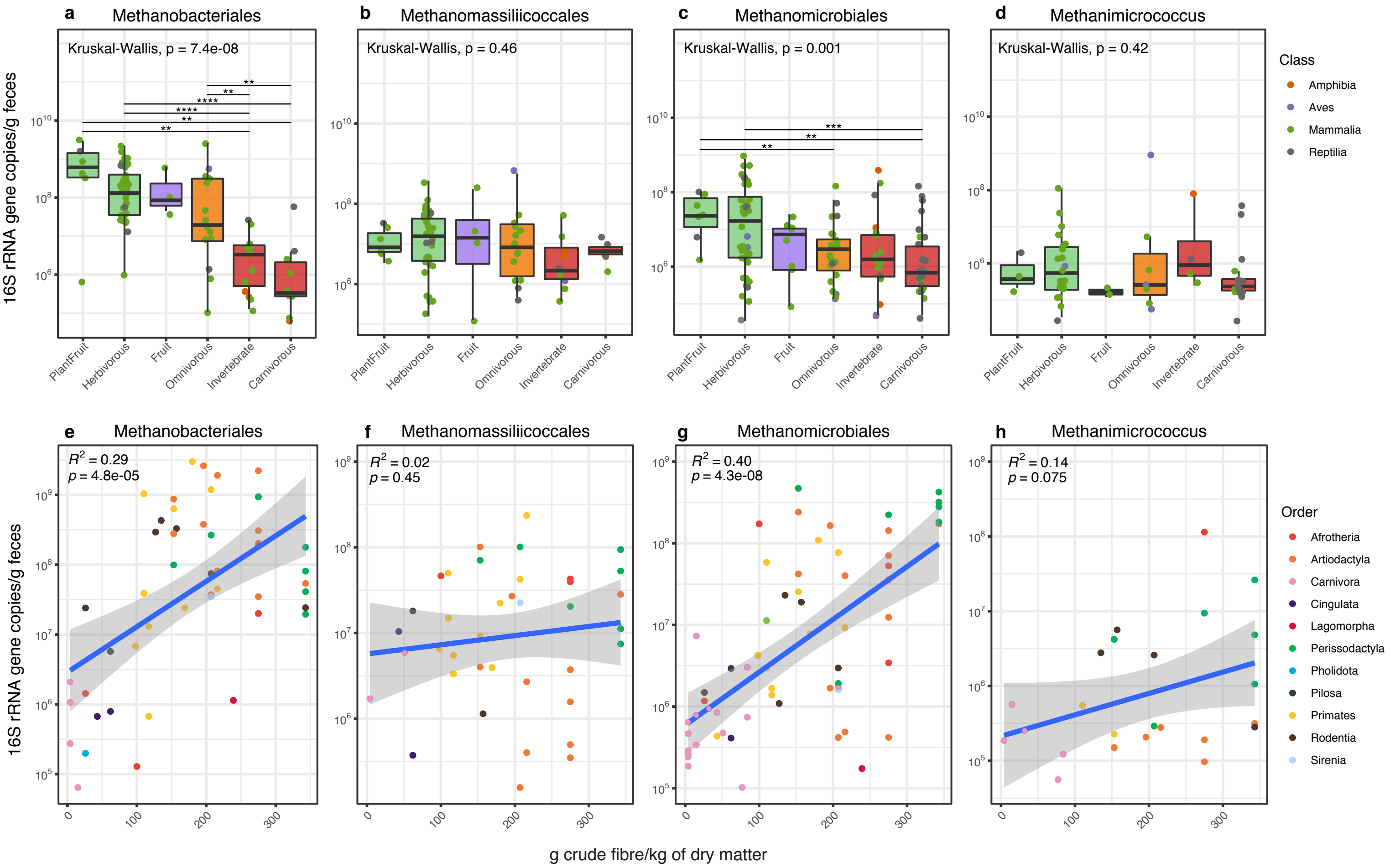

Figure S14: Influence of host diet-type and diet-fibre content on the absolute abundance of four methanogen lineages. Abundance of **a**) Methanobacteriales (n = 85), **b**) Methanomassiliicoccales (n = 73), **c**) Methanomicrobiales (n = 132) and **d**) Methanimicrococcus (n = 55) according to host diets-type. Significant differences across all groups were determined via the Kruskal-Wallis test, p < 0.05 is significant. Two-sided Wilcoxon rank sum test with continuity correction was used to determine differences between diet types **: p < 0.01; ***: p < 0.001; ****t: p < 0.0001. In the boxplots, the minima is the minimum value, maxima is the maximum values, center is the median and quartiles are shown by the box and whiskers, individual samples are shown as colored dots. The color of boxplot background in panels a-d indicates the main food type: green, leaves; purple, fruits; orange, divers; red, meat. Correlation between diet-fibre content and absolute abundance of **e**) Methanobacteriales (n = 50), **f**) Methanomassiliicoccales (n = 38), **g**) Methanomicrobiales (n = 61) and **h**) Methanimicrococcus (n = 24) in mammal species. A two-sided, squared Pearson correlation coefficient was computed to assess the relationship between values, unadjusted p-values < 0.05 were considered significant. Grey bands around the lines (panels e-h) represent the 95% confidence interval around the linear regression model.

Figure S15

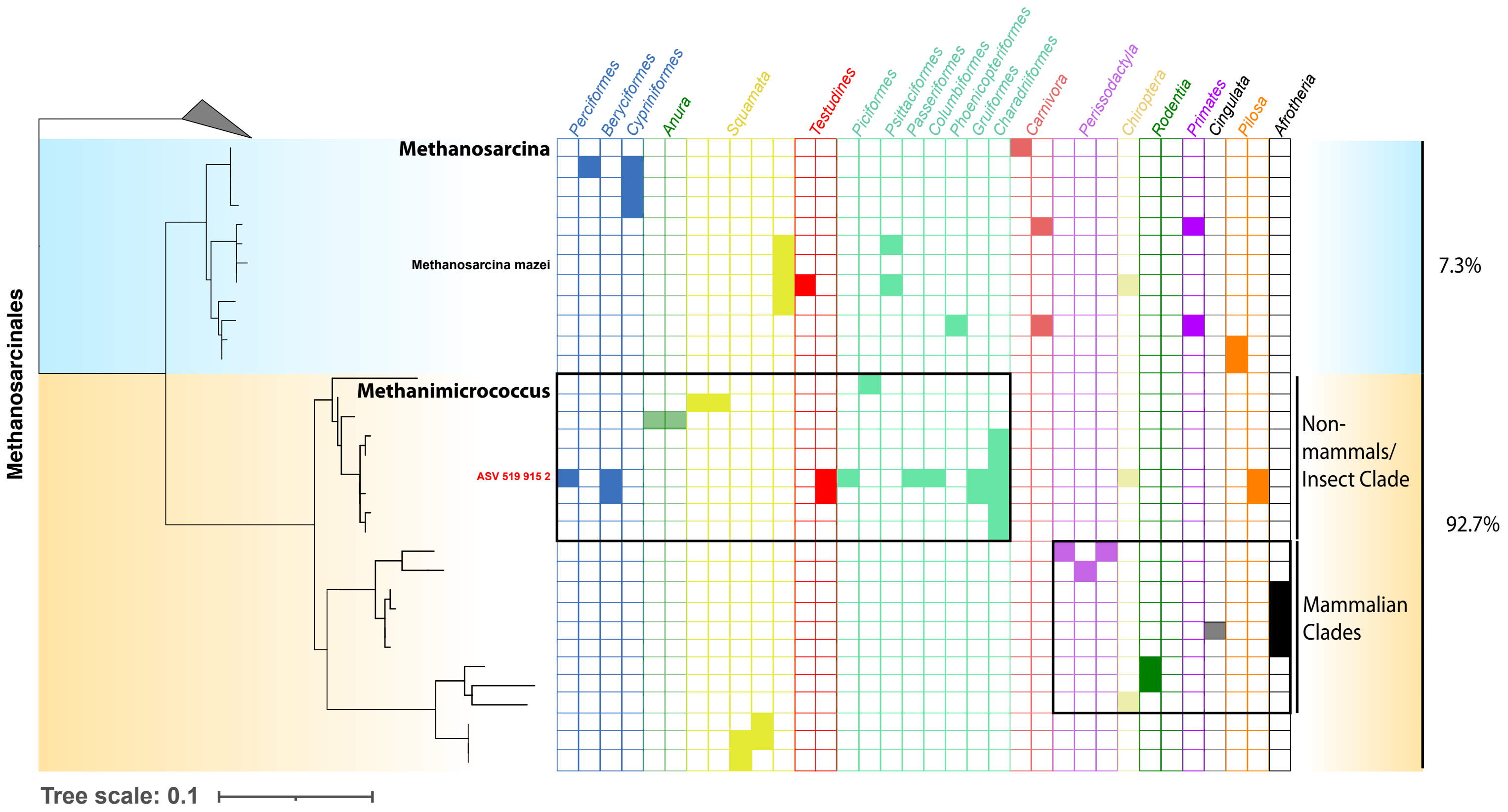

Figure S15: Distribution of Methanosarcinales ASVs among animals. The phylogenetic tree (maximum-likelihood, GTR+G4) was constructed with nearly full length 16S rRNA genes sequences from literature and the ASVs sequences from this study. For clarity, the full 16S rRNA genes from literature were then removed from the tree. Only ASVs representing more than 1% of the sequences per sample were included. Presence/absence of ASVs in animals is indicated by coloured highlighted/blank squares. Animal were gathered by orders, each with a different colour, as indicated on the top. The percentages on the right represent the proportion of reads from *Methanosarcinales* that were annotated as *Methanimicrococcus* (yellow background) and *Methanosarcina* (blue background). Black boxes highlight archaeal clades preferentially present in mammals or in non-mammals animals (and insect sequences from previous studies). Insect and mammalian clades were defined in [sup12]. Here the insect clade comprises mainly sequences from vertebrates feeding on invertebrates.

Figure S16

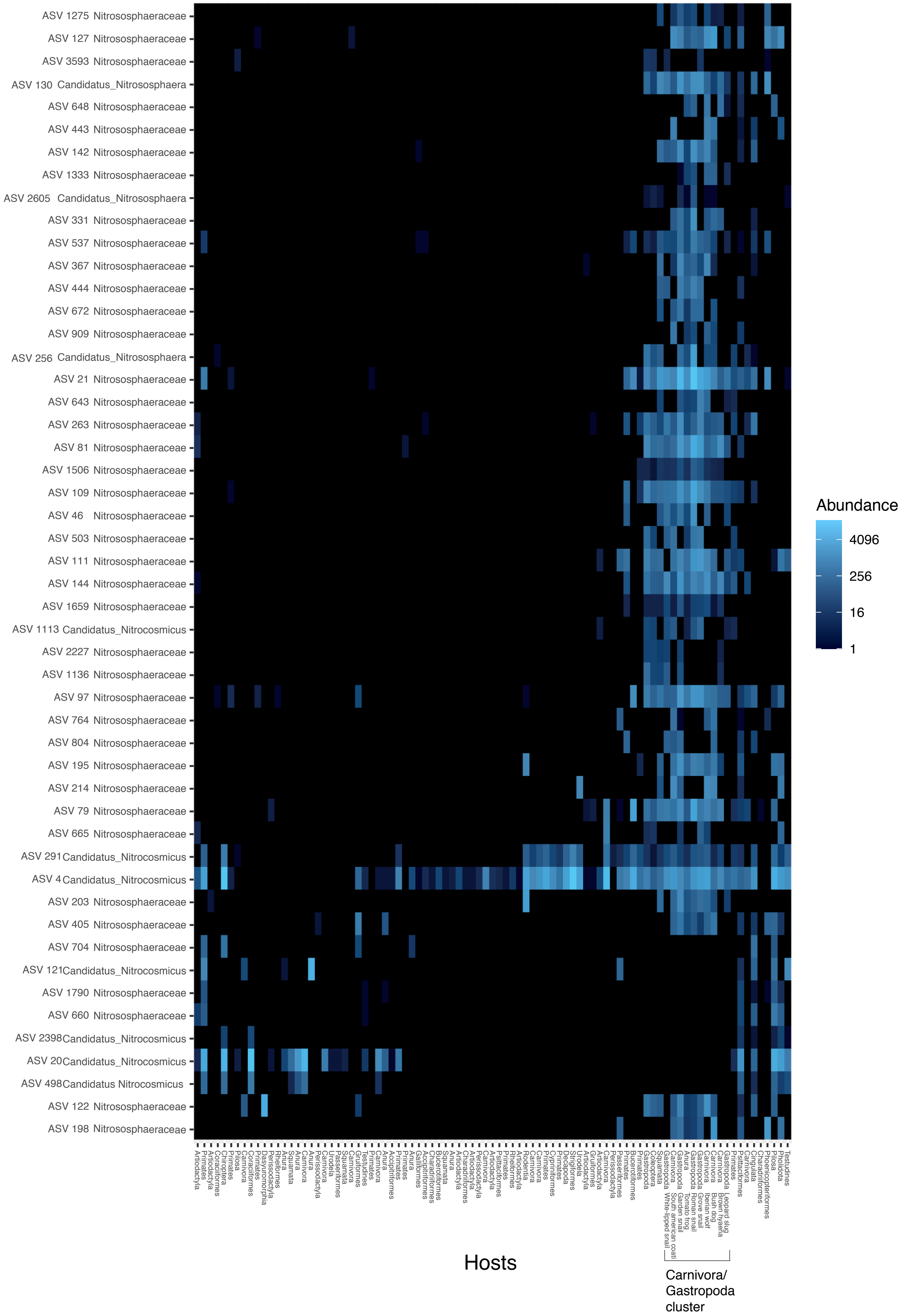

Figure S16: Unweighted UniFrac–MDS of most prevalant Thaumarchaeota ASVs (found in >5% of samples) (n=89). Animals and ASVs are clustered by the unweighted UnifFrac dissimilarity measure. Carnivora and Gastropoda host similar Thaumarchaeota communities.

Figure S17

Unweighted UniFrac - MDS Clustering of Most Prevelant Methanobacteriales ASVs in Mammals

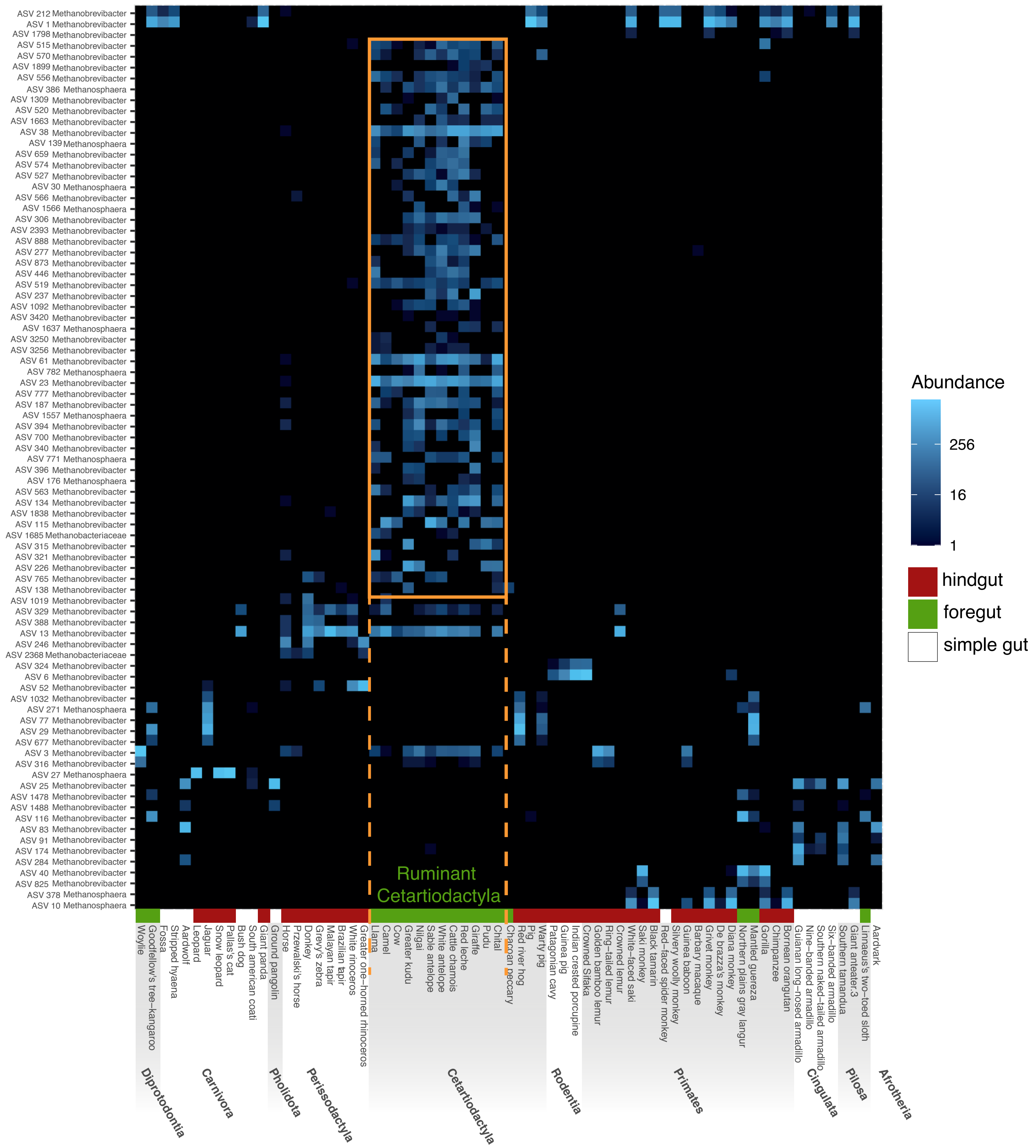

Figure S17: Unweighted UniFrac–MDS clustering of most prevalant *Methanobacteriales* ASVs in mammals (ASVs that are found in >5% of mammals (n=67; rarefied 3000 reads/sample)). Animals are organized according to mammal phylogeny, and ASVs on y-axis are clustered by the unweighted UniFrac dissimilarity measure. Most ruminant Cetartiodactyla host a distinct community of *Methanobacteriales* (orange box).

Figure S18

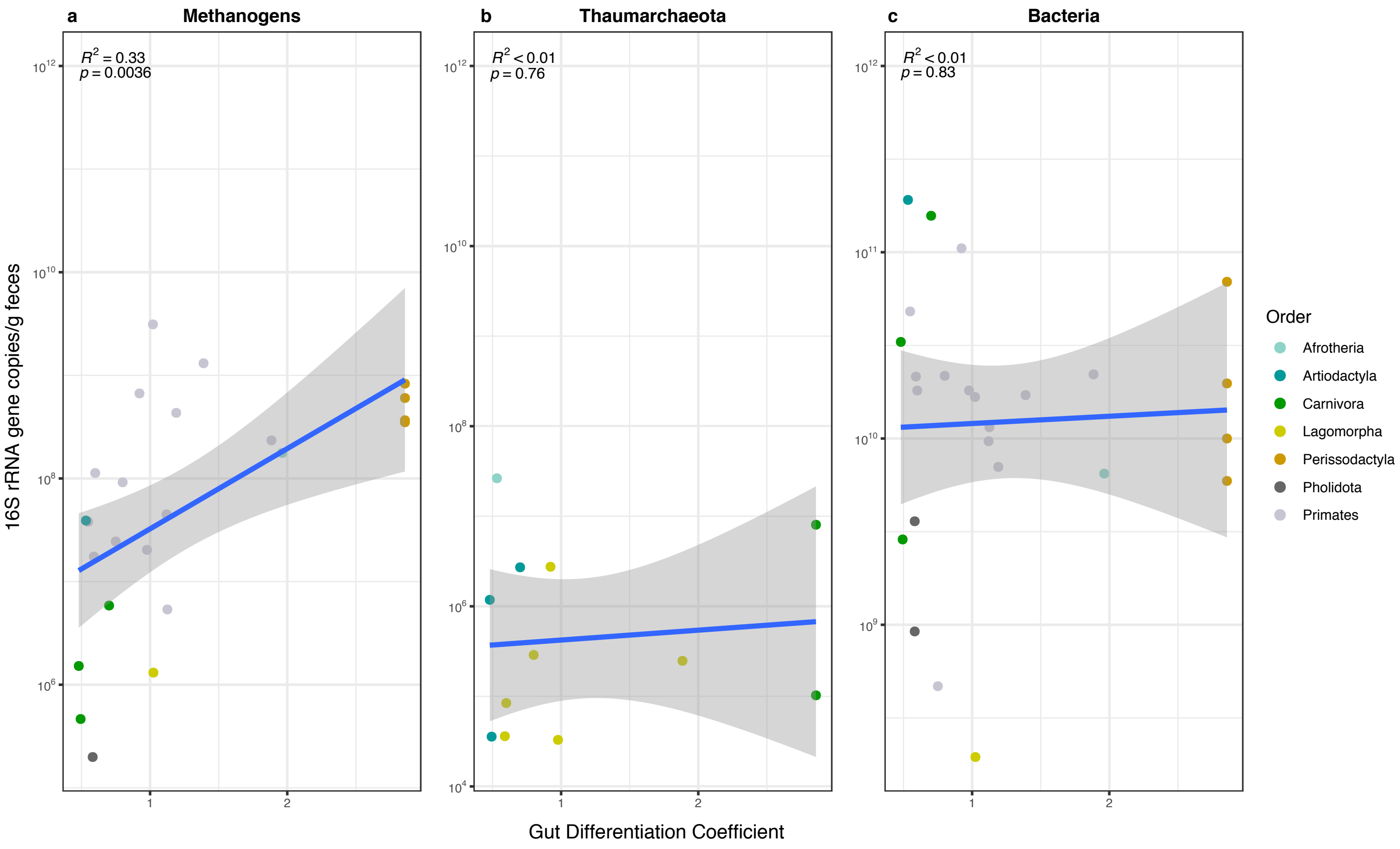

Figure S18: Correlation between gut differentiation coefficient and the absolute abundance of **a**) methanogens (n=25), **b**) Thaumarchaeota (n= 12) and **c**) bacteria (n=25) in mammals. The coefficient of gut differentiation corresponds to the surface area of the stomach, caecum, and colon, relative to the surface of the small intestine and is related to the proportion of the intestinal tract dedicated to fermentation. Two-sided, squared Pearson correlation coefficient was computed to assess the relationship between values, unadjusted p-values <0.05 were considered significant. Grey bands around the lines represent the 95% confidence interval around the linear regression model.

Figure S19

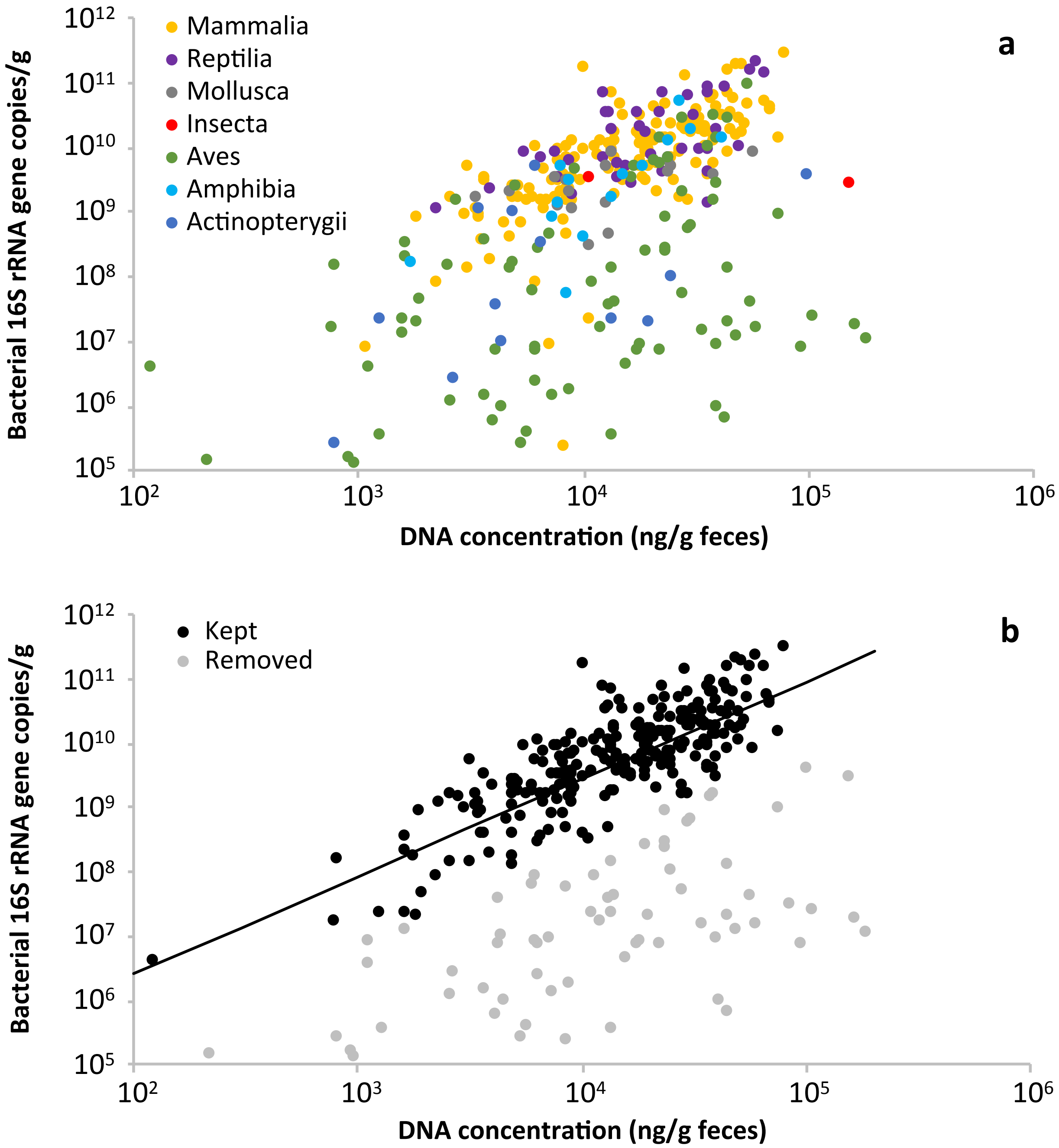

Figure S19: Comparison between DNA concentration and bacterial 16S rRNA gene copy number (n = 323 samples). **a**) Samples were labelled by animal class. **b**) A regression curve between the 16S rRNA gene copy number and the DNA concentration was determined for all samples except those from Aves (birds) and Actinopterygii (fish). For a given DNA concentration, all samples with a 16S rRNA gene copy number more than 10-fold lower than the value on the regression curve were removed from subsequent analyses of the qPCR data (those in grey). These samples are indicated in Supplementary Data 1.

Supplementary references

1. Borrel, G. *et al.* Phylogenomic data support a seventh order of methylotrophic methanogens and provide insights into the evolution of methanogenesis. *Genome Biol. Evol.* **5**, 1769–1780 (2013).
2. Söllinger, A. *et al.* Phylogenetic and genomic analysis of Methanomassiliicoccales in wetlands and animal intestinal tracts reveals clade-specific habitat preferences. *FEMS Microbiol. Ecol.* **92**, (2016).
3. Lang, K. *et al.* New mode of energy metabolism in the seventh order of methanogens as revealed by comparative genome analysis of 'Candidatus Methanoplasma termitum'. *Appl. Environ. Microbiol.* **81**, 1338–1352 (2015).
4. Raymann, K., Moeller, A. H., Goodman, A. L. & Ochman, H. Unexplored Archaeal Diversity in the Great Ape Gut Microbiome. *mSphere* **2**, e00026-17 (2017).
5. Leadbetter, J. R. & Breznak, J. A. Physiological ecology of Methanobrevibacter cuticularis sp. nov. and Methanobrevibacter curvatus sp. nov., isolated from the hindgut of the termite Reticulitermes flavipes. *Appl. Environ. Microbiol.* **62**, 3620–3631 (1996).
6. Leadbetter, J. R., Crosby, L. D. & Breznak, J. A. Methanobrevibacter filiformis sp. nov., a filamentous methanogen from termite hindguts. *Arch. Microbiol.* **169**, 287–292 (1998).
7. Hackstein, J. H. & Stumm, C. K. Methane production in terrestrial arthropods. *Proc. Natl. Acad. Sci.* **91**, 5441–5445 (1994).
8. Sprenger, W. W., Hackstein, J. H. P. & Keltjens, J. T. The energy metabolism of Methanomicrococcus blatticola: Physiological and biochemical aspects. *Antonie van Leeuwenhoek, Int. J. Gen. Mol. Microbiol.* **87**, 289–299 (2005).
9. Sprenger, W. W., Hackstein, J. H. P. & Keltjens, J. T. The competitive success of Methanomicrococcus blatticola, a dominant methylotrophic methanogen in the cockroach hindgut, is supported by high substrate affinities and favorable thermodynamics. *FEMS Microbiol. Ecol.* **60**, 266–275 (2007).
10. Brune, A. & Dietrich, C. The Gut Microbiota of Termites: Digesting the Diversity in the Light of Ecology and Evolution. *Annu. Rev. Microbiol.* **69**, 145–166 (2015).
11. Sprenger, W. W., Van Belzen, M. C., Rosenberg, J., Hackstein, J. H. P. & Keltjens, J. T. Methanomicrococcus blatticola gen. nov., sp. nov., a methanol- and methylamine-reducing methanogen from the hindgut of the cockroach Periplaneta americana. *Int. J. Syst. Evol. Microbiol.* **50**, 1989–1999 (2000).
12. Thomas, C. M., Taib, N., Gribaldo, S. & Borrel, G. Comparative genomic analysis of Methanimicrococcus blatticola provides insights into host-adaptation in archaea and the evolution of methanogenesis. *ISME Commun.* **1**, 47 (2021).
13. Lagkouvardos, I. *et al.* Sequence and cultivation study of Muribaculaceae reveals novel species, host preference, and functional potential of this yet undescribed family. *Microbiome* **7**, 1–15 (2019).
14. Ormerod, K. L. *et al.* Genomic characterization of the uncultured Bacteroidales family S24-7 inhabiting the guts of homeothermic animals. *Microbiome* **4**, 1–17 (2016).
15. Kageyama, A. & Benno, Y. Phylogenic and phenotypic characterization of some Eubacterium-like isolates from human feces: description of Solobacterium moorei Gen. Nov., Sp. Nov. *Microbiol. Immunol.* **44**, 223–227 (2000).
16. Ruaud, A. *et al.* Syntrophy via interspecies H2 transfer between christensenella and

methanobrevibacter underlies their global cooccurrence in the human gut. *MBio* **11**, e03235-19 (2020).
17. Biddle, A., Stewart, L., Blanchard, J. & Leschine, S. Untangling the genetic basis of fibrolytic specialization by lachnospiraceae and ruminococcaceae in diverse gut communities. *Diversity* **5**, 627–640 (2013).
18. Wang, W. *et al.* High-throughput sequencing reveals the core gut microbiome of Bar-headed goose (Anser indicus) in different wintering areas in Tibet. *Microbiologyopen* **5**, 287–295 (2016).
19. Manos, J. I. M. & Belas, R. The Genera *Proteus*, *Providencia*, and *Morganella*. 245–269 (2006).
20. Czerkawski, J. W. & Breckenridge, G. Determination of concentration of hydrogen and some other gases dissolved in biological fluids. *Lab. Pract.* **20**, 403–413 (1971).
21. Morgavi, D. P., Martin, C., Jouany, J. P. & Ranilla, M. J. Rumen protozoa and methanogenesis: Not a simple cause-effect relationship. *Br. J. Nutr.* **107**, 388–397 (2012).
22. Caporaso, J. G. *et al.* Global patterns of 16S rRNA diversity at a depth of millions of sequences per sample. *Proc. Natl. Acad. Sci.* **108**, 4516–4522 (2011).
23. Yu, Y., Lee, C., Kim, J. & Hwang, S. Group-specific primer and probe sets to detect methanogenic communities using quantitative real-time polymerase chain reaction. *Biotechnol. Bioeng.* **89**, 670–679 (2005).
24. Gaci, N., Chaudhary, P. P., Tottey, W., Alric, M. & Brugère, J.-F. Functional amplification and preservation of human gut microbiota. *Microb. Ecol. Health Dis.* **28**, 1308070 (2017).
25. Amann, R. I., Ludwig, W. & Schleifer, K.-H. Phylogenetic identification and in situ detection of individual microbial cells without cultivation. *Microbiol. Rev.* **59**, 143–169 (1995).
26. Gantner, S., Andersson, A. F., Alonso-S??ez, L. & Bertilsson, S. Novel primers for 16S rRNA-based archaeal community analyses in environmental samples. *J. Microbiol. Methods* **84**, 12–18 (2011).
27. Lane, D. J. 16S/23S rRNA sequencing. *Nucleic acid Tech. Bact. Syst.* 115–175 (1991).
28. Nossa, C. W. *et al.* Design of 16S rRNA gene primers for 454 pyrosequencing of the human foregut microbiome. *World J. Gastroenterol. WJG* **16**, 4135 (2010).
29. Parada, A. E., Needham, D. M. & Fuhrman, J. A. Every base matters: Assessing small subunit rRNA primers for marine microbiomes with mock communities, time series and global field samples. *Environ. Microbiol.* **18**, 1403–1414 (2016).
30. Apprill, A., McNally, S., Parsons, R. & Weber, L. Minor revision to V4 region SSU rRNA 806R gene primer greatly increases detection of SAR11 bacterioplankton. *Aquat. Microb. Ecol.* **75**, 129–137 (2015).
31. Casamayor, E. O. *et al.* Changes in archaeal, bacterial and eukaryal assemblages along a salinity gradient by comparison of genetic fingerprinting methods in a multipond solar saltern. *Environ. Microbiol.* **4**, 338–348 (2002).
32. Ametaj, B. N. *et al.* Metabolomics reveals unhealthy alterations in rumen metabolism with increased proportion of cereal grain in the diet of dairy cows. *Metabolomics* **6**, 583–594 (2010).
33. Ovreås, L., Forney, L., Daae, F. L. & Torsvik, V. Distribution of bacterioplankton in meromictic Lake Saelenvannet, as determined by denaturing gradient gel electrophoresis of PCR-amplified gene fragments coding for 16S rRNA. *Appl. Environ.*

Microbiol. **63**, 3367–3373 (1997).
34. Stahl, D. A. & Amann, R. I. Development and application of nucleic acid probes in bacterial systematics. in *Nucleic acid techniques in bacterial systematics* (eds. Stackebrandt, E. & Goodfellow, M.) 205–248 (John Wiley & Sons, 1991).
35. Steggerda, F. R. Gastrointestinal gas following food consumption. *Ann. N. Y. Acad. Sci.* **150**, 57–66 (1968).
36. Kalantar-Zadeh, K. *et al.* A human pilot trial of ingestible electronic capsules capable of sensing different gases in the gut. *Nat. Electron.* **1**, 79–87 (2018).
37. Borrel, G. *et al.* Genomics and metagenomics of trimethylamine-utilizing Archaea in the human gut microbiome. *ISME J.* **11**, 2059–2074 (2017).
38. Zhuang, G. *et al.* Biogeochemistry, microbial activity, and diversity in surface and subsurface deep-sea sediments of South China Sea. *Limnol. Oceanogr.* **64**, 2252–2270 (2019).
39. Xu, L. *et al.* Methyl-compounds driven benthic carbon cycling in the sulfate-reducing sediments of South China Sea. *Environ. Microbiol.* **23**, 641–651 (2021).
40. Zhuang, G.-C. *et al.* Multiple evidence for methylotrophic methanogenesis as the dominant methanogenic pathway in hypersaline sediments from the Orca Basin, Gulf of Mexico. *Geochim. Cosmochim. Acta* **187**, 1–20 (2016).
41. Hong, J.-K., Kim, H.-J. & Cho, J.-C. Novel PCR primers for the archaeal phylum Thaumarchaeota designed based on the comparative analysis of 16S rRNA gene sequences. *PLoS One* **9**, e96197 (2014).
42. Youngblut, N. D. *et al.* Vertebrate host phylogeny influences gut archaeal diversity. *Nat. Microbiol.* **6**, 1443–1454 (2021).
