# Supplementary material for: Factors shaping the abundance and diversity of the gut archaeome across the animal kingdom
Source: Nat Commun. 2022 Jun 10;13:3358. doi: 10.1038/s41467-022-31038-4 (PMC9187648; doi:10.1038/s41467-022-31038-4)
Supplement: Supplementary file 3 — Description of Additional Supplementary file [file 41467_2022_31038_MOESM3_ESM.docx]

File Name: **Supplementary Data 1**

Description: Sample information, animal species metadata, archaeal and bacterial abundance, archaeal ASV richness, and relative abundance of archaeal lineages. Only samples for which DNA was successfully extracted and PCR amplifications obtained were shown.

File Name: **Supplementary Data 2**

Description: Co-occurrence analyses.

First tab: ASVs were pooled into OTUs at a 97% similarity-cut off and normalized using the Archaea – Bacteria ratio determined via qPCR. Only cooccurring OTUs matching between SparCC and SPIEC-EASI algorithms were counted. Well represented groups (>6 species per order) of mammals, birds, and reptiles were all analysed. Perissodactyla and Cetartiodactyla (Ungulata) samples were analysed together to increase the robustness of the analysis. Correlation values calculated with SparCC represent two-sided Pearson correlation coefficients. Correlation were considered significant when p <0.05. For SPIEC-EASI, the minimal edge stability thresholds are indicated for each animal group.

Second tab: Summary of the archaeal OTUs showing a significant correlation/edge weight with bacteria or other archaea in both cooccurrence (SparCC and SPIEC-EASI) analyses.

File Name: **Supplementary Data 3**

Description: Detection of archaea in animal species sampled in our study (noted CT) and in Youngblut et al., ^sup42^ study (noted NY).

File Name: **Supplementary Data 4**

Description: Exact p-values for significant differences presented in the main and supplementary figures.
