# Supplementary material for: Factors shaping the abundance and diversity of the gut archaeome across the animal kingdom
Source: Nat Commun. 2022 Jun 10;13:3358. doi: 10.1038/s41467-022-31038-4 (PMC9187648; doi:10.1038/s41467-022-31038-4)
Supplement: Supplementary file 8 — Reporting Summary [file 41467_2022_31038_MOESM8_ESM.pdf]

nature portfolio

Corresponding author(s): Guillaume Borrel

Last updated by author(s): Aug 27, 2021

Reporting Summary

Nature Portfolio wishes to improve the reproducibility of the work that we publish. This form provides structure for consistency and transparency in reporting. For further information on Nature Portfolio policies, see our Editorial Policies and the Editorial Policy Checklist.

Statistics

For all statistical analyses, confirm that the following items are present in the figure legend, table legend, main text, or Methods section.

n/a	Confirmed	
☐	☒	The exact sample size (*n*) for each experimental group/condition, given as a discrete number and unit of measurement
☒	☐	A statement on whether measurements were taken from distinct samples or whether the same sample was measured repeatedly
☐	☒	The statistical test(s) used AND whether they are one- or two-sided<br>*Only common tests should be described solely by name; describe more complex techniques in the Methods section.*
☐	☒	A description of all covariates tested
☐	☒	A description of any assumptions or corrections, such as tests of normality and adjustment for multiple comparisons
☐	☒	A full description of the statistical parameters including central tendency (e.g. means) or other basic estimates (e.g. regression coefficient) AND variation (e.g. standard deviation) or associated estimates of uncertainty (e.g. confidence intervals)
☐	☒	For null hypothesis testing, the test statistic (e.g. *F*, *t*, *r*) with confidence intervals, effect sizes, degrees of freedom and *P* value noted<br>*Give P values as exact values whenever suitable.*
☒	☐	For Bayesian analysis, information on the choice of priors and Markov chain Monte Carlo settings
☒	☐	For hierarchical and complex designs, identification of the appropriate level for tests and full reporting of outcomes
☒	☐	Estimates of effect sizes (e.g. Cohen's *d*, Pearson's *r*), indicating how they were calculated

Our web collection on statistics for biologists contains articles on many of the points above.

Software and code

Policy information about availability of computer code

Data collection	No software was used for data collection
Data analysis	Statical analysis R studio and R (v3.6.0) packages: Dada2 (v1.12.1), phyloseq (v1.30.0), stats (v3.6.0); vegan (v2.5-6); Spiec.Easi (v1.1.0); ape (v5.6.1) CHNOSZ. VSEARCH (v2.17.1), BLAST (2.9.0+). Alignments: MAFFT (v7.273). Phylogeny: IQTree (1.6.12), ITOL (5).

For manuscripts utilizing custom algorithms or software that are central to the research but not yet described in published literature, software must be made available to editors and reviewers. We strongly encourage code deposition in a community repository (e.g. GitHub). See the Nature Portfolio guidelines for submitting code & software for further information.

Data

Policy information about availability of data

All manuscripts must include a data availability statement. This statement should provide the following information, where applicable:

- Accession codes, unique identifiers, or web links for publicly available datasets
- A description of any restrictions on data availability
- For clinical datasets or third party data, please ensure that the statement adheres to our policy

Sequencing data have been deposited in GenBank under the bioproject PRJNA810306. Raw phylogenetic trees (newick files) and R markdown files are available upon request.
The following databases were used in this study: SILVA Living Tree Project LTP (ltp_12_2020) https://imedea.uib-csic.es/mmg/ltp/ltp-2020/; EltonTraits database ; Animal Diversity Web https://animaldiversity.org/; Silva SSU 138 database https://www.arb-silva.de/documentation/release-138/; RDP database (11.5) http://rdp.cme.msu.edu/index.jsp.

Field-specific reporting

Please select the one below that is the best fit for your research. If you are not sure, read the appropriate sections before making your selection.

☐ Life sciences ☐ Behavioural & social sciences ☒ Ecological, evolutionary & environmental sciences

For a reference copy of the document with all sections, see nature.com/documents/nr-reporting-summary-flat.pdf

Ecological, evolutionary & environmental sciences study design

All studies must disclose on these points even when the disclosure is negative.

Study description	An archaea-centric 16S rRNA gene amplicon sequencing and quantification that targeted the gut microbiome of a diverse range of animals.
Research sample	Little is known about the composition and abundance of archaeal lineages in the gut, despite from a few host species. So we collected feacal (or gut content) samples from diverse groups of animals including - mammals, birds, reptiles, fish, amphibians, and invertebrates. Organism taxa, source and additional information on the samples are provided in Supplementary Dataset 1.
Sampling strategy	Guillaume Borrel contacted French zoological institutions and researchers to request the donation of faecal samples from animals whose intestinal archaeome is poorly known. Samples were donated by Jérôme Fuchs from the Museum National d'Histoire Naturelle (MNHN), Frédéric Delsuc (Institut des Sciences de l'Evolution, UMR5554), Alexis Lécu and Olivier Marquis from Parc Zoologique de Paris, Baptiste Mulot and Hanae Pouillevet from Zoo de Beauval, Paola Dvihally and Dominique Gitton from Parc des Mammelles, Yann Reynaud, Matthieu Pot, Gaëlle Gruel, Séverine Ferdinand from Institut Pasteur de Guadeloupe, Thomas Godoc from Aquarium de Guadeloupe, Patrick Buisson and Sabine Collin at Palais de la Découverte, Jérôme Contignac and David Luis Garcias Warner from Nabau Projects/Grupo Atrox, Aude Bourgeois from Jardin des Plantes de Paris, Jérémy Sauvanet, Anaïs Tibi and Johnatan Aparicio. Faecal samples (n = 341) were stored at -20°C until DNA extraction. No sample size calculation was performed and we collected all available samples. Main objectives of the study were fulfilled using the collected samples, such as highlighting several factors driving the abundance and diversity of archaea in the animal gut.
Data collection	Courtney Thomas collected host metadata from the EltonTraits database, the Animal Diversity Web database and the literature. Other information about the samples was provided by the specialists who donated the samples. All data were recorded in spreadsheet.
Timing and spatial scale	Sample donation began in July 2018 and concluded in May 2019. DNA extractions were preformed as samples were received and concluded in July 2019. Quantitative PCR analyses began in May 2019. Sequencing was preformed in December of 2019. Analysis of the data began in February 2020.<br>Our samples were primarily collected in Mainland France but also in French Guadalupe, the USA, and South Africa.
Data exclusions	No sample was excluded from 16S rRNA gene amplicon sequencing or quantitative PCR. Samples with a low bacterial 16S rRNA gene concentration compared to the DNA concentration were removed from qPCR analyses.
Reproducibility	Reproducibility was addressed by comparing data obtained with different approaches on the same samples. For example, the abundance of all archaea correlates with the sum of the abundance of individual archaeal lineages quantified independently.
Randomization	No randomization was applied in our study as we have sampled only one individual for the vast majority of the animal species and never more than 5 individuals.
Blinding	Blinding was not used in our study as it is not a clinical trial.

Did the study involve field work? ☐ Yes ☒ No

Reporting for specific materials, systems and methods

We require information from authors about some types of materials, experimental systems and methods used in many studies. Here, indicate whether each material, system or method listed is relevant to your study. If you are not sure if a list item applies to your research, read the appropriate section before selecting a response.

Materials & experimental systems

n/a	Involved in the study	
☒	☐	Antibodies
☒	☐	Eukaryotic cell lines
☒	☐	Palaeontology and archaeology
☐	☒	Animals and other organisms
☒	☐	Human research participants
☒	☐	Clinical data
☒	☐	Dual use research of concern

Methods

n/a	Involved in the study	
☒	☐	ChIP-seq
☒	☐	Flow cytometry
☒	☐	MRI-based neuroimaging

Animals and other organisms

Policy information about studies involving animals; ARRIVE guidelines recommended for reporting animal research

Laboratory animals	Laboratory animals were not used in this study.
Wild animals	Wild bird samples were collected from deceased birds (frozen right after death) by Jérôme Fuch, a specialist at the Museum National d'Histoire Naturelle. Frederic Delusc donated samples from wild armadillos, hyraxes, pangolins, and aardwolves collected for previous studies. Wild snails were caught by hand and kept captive for for 24 hrs for feces collections and were released in their environment of origin. Species taxonomy is provided in Supplementary Dataset 1.
Field-collected samples	All other samples were collected from captive animals housed in zoological facilities within France. No data was acquired on their environment.
Ethics oversight	After consultation with the corresponding Ethics Committee in Animal Experimentation our study got direct clearance since the samples that were used for this project were collected either from species or by methods that are not in the scope of the current regulation protecting the animals used for scientific purposes (European Directive 2010/63/EU, and French Decree 2013-118).

Note that full information on the approval of the study protocol must also be provided in the manuscript.
